# The Burden of Childhood Asthma by Age Group, 1990–2019: A Systematic Analysis of Global Burden of Disease 2019 Data

**DOI:** 10.3389/fped.2022.823399

**Published:** 2022-02-16

**Authors:** Daoqi Zhang, Jinxin Zheng

**Affiliations:** ^1^Department of Internal Medicine Teaching and Research Section, Xuancheng Vocational and Technical College, Xuancheng, China; ^2^Department of Nephrology, Ruijin Hospital, Shanghai Jiao Tong University School of Medicine, Shanghai, China

**Keywords:** disability-adjusted life years (DALYs), sociodemographic index (SDI), childhood asthma, body-mass index, risk factors

## Abstract

**Background:**

Asthma is a common respiratory disease in children. We aimed to update information about the incidence and mortality and disability-adjusted life years (DALYs) of childhood asthma and provide evidence-based recommendations for childhood asthma prevention.

**Methods:**

Data were obtained from the Global Burden of Disease (GBD) study, which was conducted from 1990 to 2019 in 204 countries. First, we estimated incidence, mortality and DALY rates of childhood asthma using a Bayesian meta-regression model. Second, we analyzed the relationship between the sociodemographic index (SDI) and DALYs in different age groups. Third, we studied changes in trends of the age-standardized DALY rate between 1990 and 2019 based on age group, SDI, and risk factors.

**Results:**

Globally, the number of deaths due to childhood asthma and the incidence and DALY rates were 12.9 thousand (95% UI 10.6 to 15.7), 22 million (95% UI 15 to 31), and 5.1 million (95% UI 3.4 to 7.5) in 2019, decreasing by 65.1% (95% UI 47.6 to 72.4), 5.3% (95% UI 2.6 to 8.8) and 30% (95% UI 18 to 41) from those in 1990, respectively. With the exception of high-SDI regions, the age-standardized DALY rate in all age groups in all SDI regions declined. In 2019, the age-standardized DALY rate in 1- to 4-year-old individuals was highest in low-SDI regions and that of 5- to 19-year-old individuals was highest in high-SDI regions. In contrast to low-SDI regions, individuals in high-SDI regions had a higher risk of DALYs due to asthma, except in those aged 1 to 4 years. A high body mass index (BMI) was a stronger risk factor than occupational asthmagens for childhood asthma.

**Conclusion:**

Our findings provide insight into asthma prevention and treatment through the identification of key factors related to childhood asthma. Based on the data available, different risk factors according to age group and region/country suggest different prevention strategies, which is key for preventing childhood asthma.

## Introduction

Childhood asthma is a major common chronic respiratory illness characterized by wheezing, coughing, shortness of breath and airflow limitation, which affects daily life (1, 2). The pathogenesis of childhood asthma includes complex interactions involving physics, chemistry, pharmacology, and immunology, resulting in excessive mucus secretion, bronchial edema and spasm, and scar remodeling (3). Although inhaled corticosteroids can control the symptoms of asthma, some children with persistent asthma still experience severe complications and lung dysfunction (4). Due to a lack of understanding of health care for childhood asthma, reduced treatment efficacy and incomplete control of lung damage may occur (5). Childhood asthma imposes the highest disability burden, causing almost 13.8 million days of absence from school in the United States in 2013. Furthermore, children with asthma need to receive psychological support because asthma can lead to a lower education level and early dropout from school (6, 7). In this study, we estimated the disability burden of childhood asthma based on disability-adjusted life years (DALYs) from 1990 to 2019 by region and country as well as by age.

Globally, comorbidities of childhood asthma include allergic rhinitis, loss of lung function and mental illness (8, 9), and the burden of childhood asthma is substantial in high-income countries. Overall, medical expenditures and DALYs due to severe asthma are high. Establishing a model of health care management that will reduce the number of DALYs and costs of childhood asthma is recommended (10, 11). Children with asthma need formal monitoring and disease management to reduce the number of DALYs and to control symptoms. The lack of comprehensive health care policies is likely one of the main reasons for the increasing incidence of DALYs due to childhood asthma. Research on environmental factors, lifestyle behaviors, dietary habits, and other health-related risk factors will guide effective prevention of the occurrence of childhood asthma. Nevertheless, improving the level of medical care requires substantial knowledge about how to prevent diseases and associated risk factors that are harmful and lead to disability. The Global Burden of Disease (GBD) study dataset is useful for risk factor quantification, as it contains reliable data on childhood asthma for 1990–2019; the findings of data analyses can help to inform regional and national health policies (12).

In this study, we estimated the incidence, mortality and DALY rates of childhood asthma using GBD data from 1990 to 2019; the data were stratified by age, sex, sociodemographic index (SDI), region and country. We also discuss detailed information about risk factors for DALYs due to childhood asthma and present the relationship between age group and sociodemographic index (SDI) at DALYs. The results offer evidence for informing healthcare management of childhood asthma.

## Methods

### Data Sources

Childhood asthma is a chronic lung disease caused by allergic or hypersensitivity reactions and characterized by bronchospasms and dyspnea (13). Childhood asthma in the GBD study was defined as a diagnosis by a doctor and the presence of International Classification of Diseases, 10th edition (ICD-10), codes J45 and J46. The methods of the GBD study have been widely reported, including data for incidence, death and DALY rates for 1990 to 2019, as based on systematic papers, unpublished reports and surveys, and health service data from the USA. Detailed information on childhood asthma can be found at http://ghdx.healthdata.org/gbd-results-tool.

The SDI is a summary measure that reflects sociodemographic development, including local income, average educational attainment, and total fertility rates (14). SDI values range from 0 (lowest income, lowest educational attainment, and highest fertility rate) to 1 (highest income, highest educational attainment, and lowest fertility rate) (15–17). The 204 countries and territories in the GBD study were classified into high-, high-middle-, middle-, low-middle-, and low-SDI regions. The cutoff values used to determine quintiles for analysis were computed using country-level estimates of the SDI for 2019, excluding countries with populations <1 million.

### Statistical Analysis

The standardized methods of GBD 2019 have been published by the GBD team and extensively reported elsewhere. Incidence, mortality, and DALY rates for childhood asthma for 204 countries and territories from 1990 to 2019 were estimated based on age and sex using a Bayesian meta-regression model in DisMod-MR 2.1. During data processing, the mean of 1000 draws was generated for all reported data, and the 2.5th and 97.5th centiles of the ordered draw represent 95% uncertainty intervals (UIs).

A linear regression model was constructed to analyze the association between year and the age-standardized incidence, mortality, and DALY rates for childhood asthma separately. Using a regression model, we scaled the numbers and age-standardized rates to make them comparable, without regard to the measurement units used in the process. Then, a generalized linear model was fitted by a Gaussian function, and the year coefficient was extracted to measure the strength and direction of the time trend. The corresponding 95% confidence interval (CI) of the year coefficient was acquired from this linear regression model. Associations of age-standardized incidence, mortality, and DALY rates with the SDI for 204 countries and territories and 21 GBD regions were evaluated by smoothing spline models (18). We applied R software V.4.0.2 to estimate incidence, mortality, and DALY rates and numbers from the GBD dataset.

## Results

### Global Burden

In 2019, 12.9 thousand (95% UI 10.6 to 15.7 thousand) children died from asthma. From 1990 to 2019, the age-standardized mortality rate decreased significantly by 65.1% (95% UI 47.6 to 72.4) to 0.5 per 100 000 (95% UI 0.4 to 0.6). The greatest decrease in the age-standardized mortality rate was in the high-middle-SDI group, and the number of incident childhood asthma cases was estimated to be nearly 22 million (95% UI 15 to 31 million). From 1990 to 2019, the global age-standardized incidence rate of childhood asthma decreased by 5.3% (95% UI 2.6 to 8.8%) to 876.0 per 100 000 (95% UI 599.7 to 1212.3). Rapid increases in age-standardized incidence rates were observed in the high-SDI and high-middle-SDI groups (Tables 1, 2). DALYs due to childhood asthma in 2019 were estimated to be 5.1 million (95% UI 3.4 to 7.5 million). Between 1990 and 2019, age-standardized DALY rates decreased substantially by 30% (95% UI 18 to 41%) to 196.62 (132.71 to 291.02). A rapid increase in the age-standardized incidence was observed in only the high-SDI group (Tables 1, 2).

**Table 1:** The burden of childhood asthma by age group, sex and SDI in 1990 and 2019.

	**Deaths (95% UI)**		**Incidence (95% UI)**		**DALYs (95% UI)**	
	**1990 cases (thousands)**	**2019 cases (thousands)**	**1990 cases (millions)**	**2019 cases (millions)**	**1990 cases (millions)**	**2019 cases (millions)**
**Age (years)**						
1~4	20.44 (11.75 to 26.60)	6.10 (4.62 to 7.98)	10.16 (6.46 to 15.38)	10.00 (6.28 to 15.28)	2.67 (1.76 to 3.57)	1.40 (0.95 to 2.10)
5~9	4.10 (3.19 to 4.85)	1.70 (1.40 to 2.05)	5.56 (2.81 to 9.36)	6.42 (3.27 to 10.83)	1.60 (1.03 to 2.51)	1.49 (0.88 to 2.48)
10~14	3.41 (2.90 to 3.82)	2.11 (1.79 to 2.51)	3.13 (1.42 to 5.00)	3.77 (1.73 to 6.00)	1.16 (0.77 to 1.70)	1.22 (0.76 to 1.86)
15~19	4.56 (3.76 to 5.21)	2.97 (2.54 to 3.43)	2.17 (1.27 to 3.32)	2.40 (1.41 to 3.66)	0.99 (0.69 to 1.41)	0.95 (0.63 to 1.44)
**Sex**						
Male	16.48 (12.06 to 20.65)	6.54 (5.45 to 7.94)	11.33 (7.79 to 15.79)	12.30 (8.35 to 17.21)	3.40 (2.49 to 4.72)	2.77 (1.85 to 4.15)
Female	16.02 (8.91 to 21.00)	6.34 (4.97 to 8.40)	9.69 (6.80 to 13.35)	10.30 (7.12 to 14.01)	3.02 (2.04 to 4.16)	2.30 (1.55 to 3.36)
**Sociodemographic factor**						
Global	32.51 (22.79 to 39.65)	12.88 (10.59 to 15.68)	21.03 (14.58 to 29.22)	22.59 (15.47 to 31.27)	6.42 (4.62 to 8.86)	5.07 (3.42 to 7.51)
High SDI	0.86 (0.79 to 0.91)	0.31 (0.28 to 0.34)	3.55 (2.47 to 4.92)	3.63 (2.57 to 4.78)	0.77 (0.49 to 1.19)	0.75 (0.47 to 1.15)
High-middle SDI	1.71 (1.30 to 2.03)	0.29 (0.25 to 0.36)	3.56 (2.42 to 5.06)	12.62 (8.64 to 17.91)	0.76 (0.50 to 1.15)	0.53 (0.32 to 0.86)
Middle SDI	10.14 (7.29 to 12.11)	2.87 (2.40 to 3.34)	7.02 (4.79 to 9.82)	6.37 (4.31 to 8.89)	2.06 (1.48 to 2.87)	1.39 (0.90 to 2.12)
Low-middle SDI	10.76 (7.42 to 13.43)	3.28 (2.69 to 3.98)	3.92 (2.78 to 5.38)	4.01 (2.72 to 5.58)	1.57 (1.14 to 2.05)	0.99 (0.68 to 1.45)
Low SDI	8.98 (5.40 to 11.98)	6.10 (4.58 to 8.18)	2.95 (2.14 to 4.01)	5.03 (3.50 to 6.87)	1.25 (0.87 to 1.63)	1.41 (1.00 to 1.97)

*UI, uncertainty interval; SDI, sociodemographic index*.

**Table 2:** Percent change in age-standardized rates of childhood asthma by age group, sex and SDI, 1990–2019.

	**Deaths (95% UI)**		**Incidence (95% UI)**		**DALYs (95 %UI)**	
	**2019 Age-Standardized rates (per 1,00,000)**	**Percent change in age-standardized rates, 1990–2019**	**2019 Age-Standardized rates (per 1,00,000)**	**Percent change in age-standardized rates, 1990–2019**	**2019 Age-Standardized rates (per 1,00,000)**	**Percent change in age-standardized rates, 1990–2019**
**Age (years)**						
1~4	1.2 (0.9 to 1.5)	−71.8 (−79.6 to −49.5)	1884.6 (1183.7 to 2879.0)	−7.2 (−11.4 to −4)	264.6 (179.1 to 394.6)	−50.5 (−63.2 to −29)
5~9	0.3 (0.2 to 0.3)	−63.0 (−69.6 to −48.8)	980.3 (500.0 to 1654.7)	3.1 (7.1 to −14.1)	228.2 (134.9 to 379.3)	−16.6 (−24.9 to −9.9)
10~14	0.3 (0.3 to 0.4)	−48.3 (−55.1 to −37.5)	587.0 (268.8 to 934.6)	0.5 (4.1 to −1.8)	189.6 (118.6 to 290.2)	−12.1 (−18.4 to −7.1)
15~19	0.5 (0.4 to 0.6)	−45.3 (−52.0 to −35.4)	387.9 (227.9 to 590.7)	−7.1 (−3.5 to −10.8)	154.1 (102.3 to 231.7)	−18.8 (−25.9 to −13.2)
**Sex**						
Male	0.5 (0.4 to 0.6)	−65.2 (−72.0 to −49.2)	925.1 (628.4 to 1355.7)	−4.9 (−8.5 to −2.0)	208.64 (138.84 to 312.52)	−28.5 (−39.2 to −17.3)
Female	0.5 (0.4 to 0.7)	−64.9 (−75.0 to −26.7)	874.14 (569.38 to 1203.57)	−5.8 (−9.1 to −2.9)	183.84 (124.03 to 268.95)	−32.5 (−45.4 to −12.2)
**Sociodemographic factor**						
Global	0.5 (0.4 to 0.6)	−65.1 (−72.4 to −47.6)	876.01 (599.68 to 1212.28)	−5.3 (−8.8 to −2.6)	196.62 (132.71 to 291.02)	−30 (−41 to −18)
High SDI	0.1 4 (0.12 to 0.15)	−62.5 (−65.3 to −57.2)	1642.81 (1165.5 to 2163.38)	8 (1 to 16)	338.46 (212.04 to 520.44)	3 (−3 to 9)
High-Middle SDI	0.09 (0.08 to 0.11)	−79.0 (−83.3 to −70.5)	873.41 (583 to 1230.22)	1 (−4 to 5)	162.21 (97.71 to 261.99)	−13 (−23 to −6)
Middle SDI	0.39 (0.45 to 0.33)	−70.5 (−76.5 to −58.8)	864.82 (584.92 to 1206.9)	−6 (−9 to −3)	188.38 (122.36 to 287.65)	−30 (−42 to −18)
Low-Middle SDI	0.47 (0.39 to 0.57)	−75.0 (−80.8 to −61.6)	577.19 (391.49 to 803.14)	−16 (−22 to −11)	142.23 (97.48 to 208.99)	−48 (−59 to −34)
Low SDI	1.02 (0.77 to 1.37)	−66.4 (−76.1 to −66.4)	842.9 (585.72 to 1150.93)	−16 (−21 to −11)	236.12 (167.92 to 329.67)	−44 (−56 to −34)

*UI, uncertainty interval; SDI, sociodemographic index*.

In 2019, 6.5 thousand (95% UI 5.4 to 7.9 thousand) males and 6.3 thousand (95% UI 5.0 to 8.4 thousand) females died from childhood asthma. From 1990 to 2019, the age-standardized mortality rates were similar between males, at 0.5 per 100 000 (95% UI 0.4 to 0.6), and females, at 0.5 per 100 000 (95% UI 0.4 to 0.7). Childhood asthma affected almost 12 million (95% UI 8 to 17 million) males and almost 10 million (95% UI 7 to 14 million) females in 2019. From 1990 to 2019, the age-standardized incidence rate was higher in males, with a decrease of 4.9% (95% UI 2.0 to 8.5%) to 925.1 per 100 000 (95% UI 628.4 to 1355.7), than in females, with a decrease of 5.8% (95% UI 2.9 to 9.1) to 874.14 per 100 000 (95% UI 569.4 to 1203.6). DALYs due to childhood asthma were higher in males, at 2.8 million (95% UI 1.8 to 4.2 million), than in females, at 2.3 million (95% UI 1.6 to 3.4 million). Similarly, from 1990 to 2019, age-standardized DALYs were higher in males, with a decrease of 28.5% (95% UI 17.3 to 39.2%) to 208.64 per 100 000 (95% UI 138.84 to 312.52), than in females, with a decrease of 32.5% (95% UI 12.2 to 45.4%) to 183.8 per 100 000 (95% UI 124.03 to 268.95) (Tables 1, 2).

**Figure 1:**
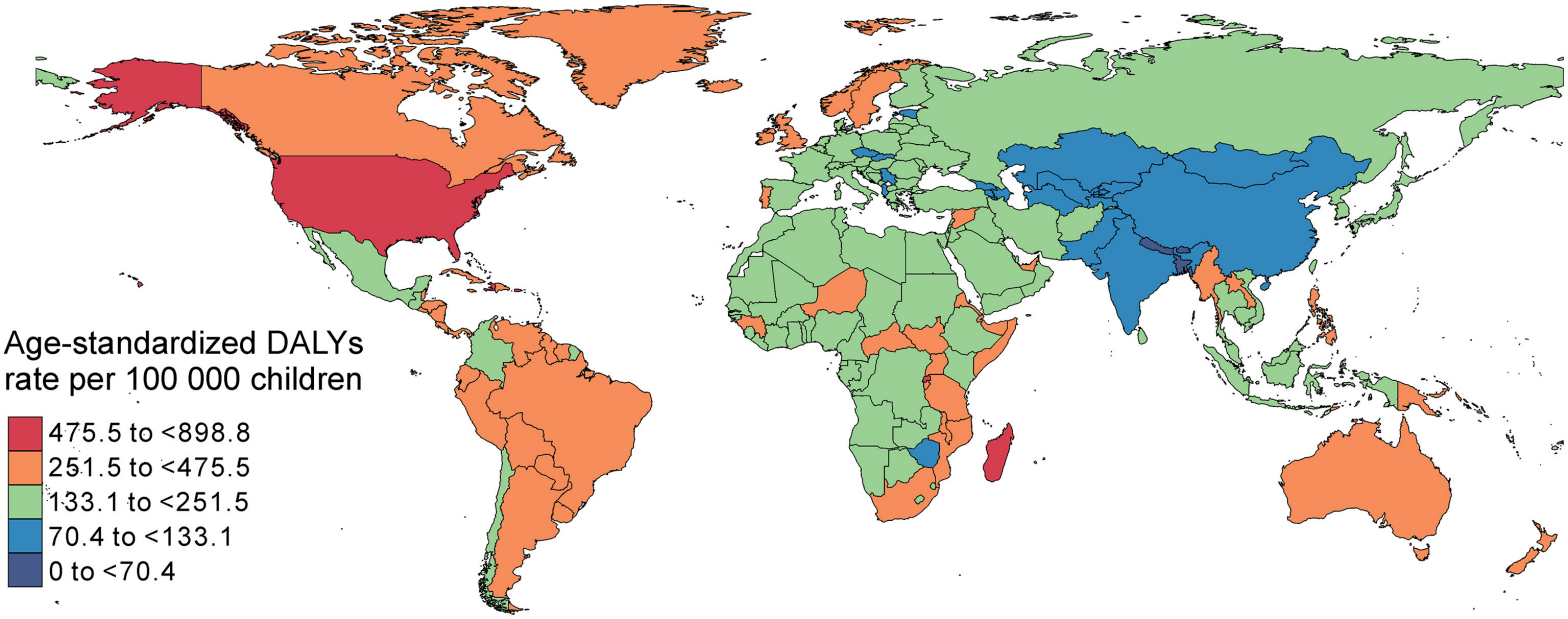
Age-standardized DALYs due to childhood asthma per 100 000 children by country, both sexes, 2019. DALYs, disability adjusted life years.

**Figure 2:**
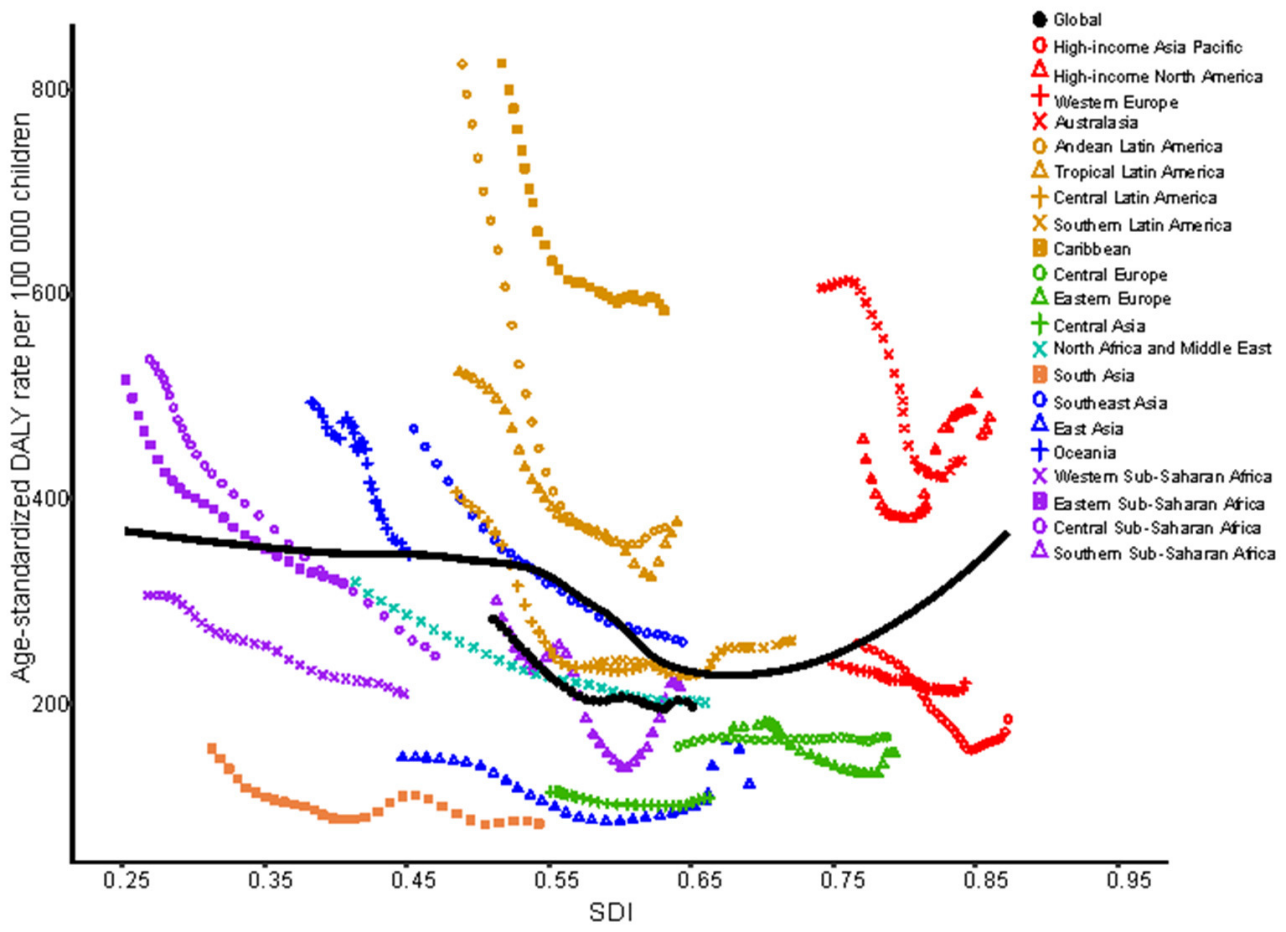
Age-standardized DALYs due to childhood asthma by 21 GBD regions and expected values by SDI, 1990–2019. DALYs, disability adjusted life years; SDI, sociodemographic index.

Globally, age-standardized DALYs were estimated to be higher than 500 per 100 000 children in some countries, such as Haiti, the United States of America, Puerto Rico and Madagascar, and lower than 100 per 100 000 children in others, such as Nepal, Bangladesh, Bhutan, Pakistan, Kazakhstan, India and Tajikistan (Figure 1). Regarding region, age-standardized DALYs due to asthma in 2019 were highest in the Caribbean [584.2 (95% UI 387.7 to 824.9) per 100 000] and high-income regions of North America [510.3 (95% UI 320.8 to 765.9) per 100 000]. Conversely, age-standardized DALYs were lowest in South Asia [82.1 (95% UI 55.4 to 120.7) per 100 000] (Figure 2). Details of the data by country, region and the percentage change in the age-standardized incidence and DALY rates between 1990 and 2019 are shown in Supplementary Table 1.

### The Burden in the 1- 4-Year-Old Age Group

The number of deaths from childhood asthma in 2019 was highest in the 1- to 4-year-old group (6.1 thousand (95% UI 4.6 to 8.0 thousand), 47.4%). The percent change in the age-standardized mortality rate (from 1990 to 2019) in the 1- to 4-year-old group declined sharply by 71.8% (95% UI 49.5 to 79.6). Moreover, 10.0 million (95% UI 6.2 to 15.3 million) children aged 1 to 4 years appeared to have asthma, and almost 44.2% of the total number of children had asthma. The percent change in the standardized incidence rate in the 1- to 4-year-old group (1990 to 2019) decreased by 7.2% (95% UI 4.0 to 11.4). The DALY rate in patients aged 1 to 4 years old was ~1.4 million (95% UI 1.0 to 2.1). The percent change in the age-standardized DALY rate (from 1990 to 2019) was notably reduced by 50.5% (95% UI 29.0 to 63.2) (Tables 1, 2).

**Figure 3:**
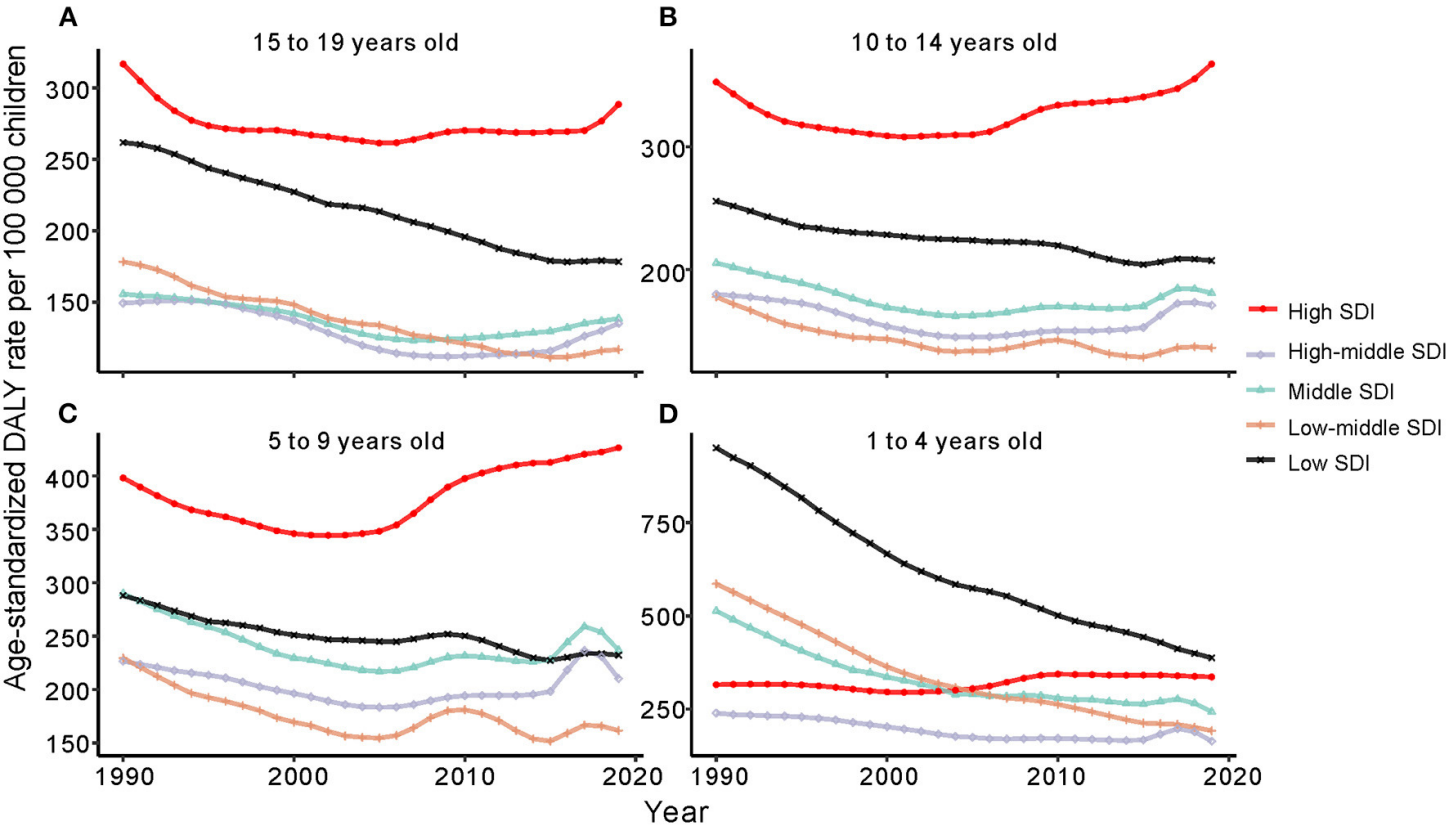
Change trends of age-standardized DALYs due to childhood asthma by age group from 1990 to 2019. **(A)** Change trends of age-standardized DALYs in 15 to 19 year olds. **(B)** Change trends of age-standardized DALYs in 10 to 14 year olds. **(C)** Change trends of age-standardized DALYs in 5 to 9 year olds. **(D)** Change trends of age-standardized DALYs in 1 to 4 year olds. DALYs, disability adjusted life years.

**Figure 4:**
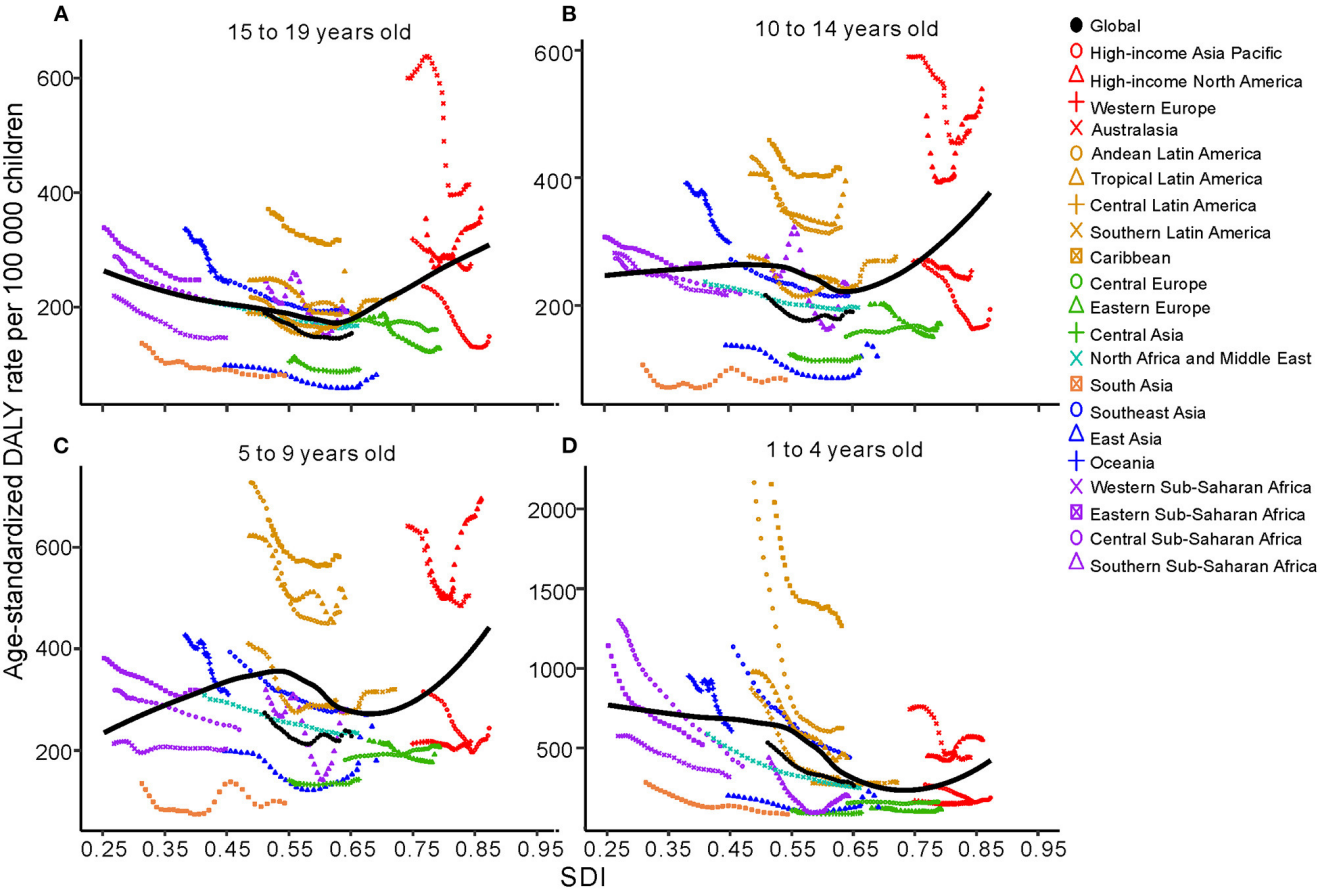
Age-standardized DALYs due to childhood asthma by 21 GBD regions and different age groups, 1990–2019; the black line represents expected values by SDI. **(A)** Change trends of age-standardized DALYs for regions by SDI in 15 to 19 years olds. **(B)** Change trends of age-standardized DALYs for regions by SDI in 10 to 14 years olds. **(C)** Change trends of age-standardized DALYs for regions by SDI in 5 to 9 year olds. **(D)** Change trends of age-standardized DALYs for regions by SDI in 1 to 4 year olds. DALYs, disability adjusted life years; SDI, sociodemographic index.

**Figure 5:**
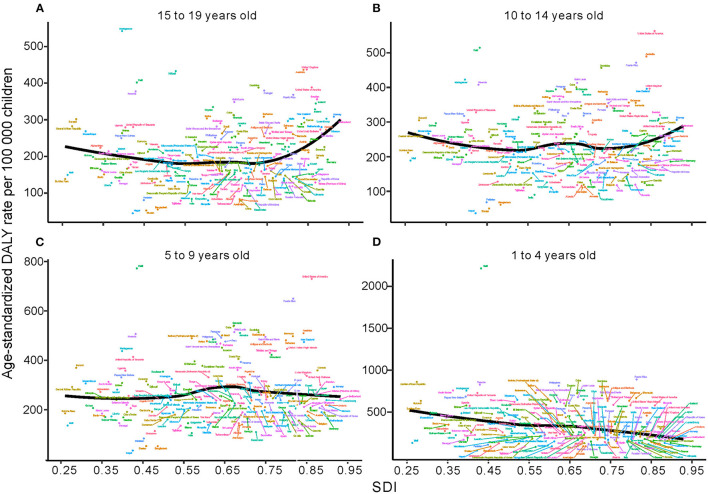
Age-standardized DALYs due to childhood asthma by country and SDI, 2019; the black line represents expected values. **(A)** Change trends of age-standardized DALYs by country and SDI in 15 to 19 year olds. **(B)** Change trends of age-standardized DALYs by country and SDI in 10 to 14 year olds. **(C)** Change trends of age-standardized DALYs by country and SDI in 5 to 9 year olds. **(D)** Change trends of age-standardized DALYs by country and SDI in 1 to 4 year olds. DALYs = disability adjusted life years, SDI, sociodemographic index.

From 1990 to 2019, age-standardized DALYs in the 1- to 4-year-old group in low-SDI, middle-SDI and low-middle-SDI regions dropped sharply, whereas that in the high-SDI region increased gradually; moreover, there was no obvious decrease in the high-middle-SDI region as a whole. The highest age-standardized DALY rate in 2019 was in the low-SDI region; the lowest DALY rate was in the high-middle SDI region (Figure 3A). At the regional level, there was an association between the age-standardized DALY rate and SDI. First, the age-standardized DALY rate was highest at an SDI of ~0.25, and it steadily declined until an SDI of 0.55. Second, age-standardized DALY rates dropped sharply from an SDI of 0.55 to an SDI of 0.75. Last, age-standardized DALY rates increased with SDIs higher than 0.75 (Figure 4A). At the national level, age-standardized DALY rates declined gradually with increasing SDI (Figure 5A).

### The Burden in the 5- to 9-Year-Old Age Group

The number of deaths due to childhood asthma in the 5- to 9-year-old group in 2019 was nearly 13.2% (1.7 thousand) overall. The percent change in the age-standardized mortality rate (from 1990 to 2019) decreased by 63.0% (95% UI 48.8 to 69.6%), and nearly 28.4% (6.4 million) of all children had asthma. The percent change in the standardized incidence rate increased by 3.1% from 1990 to 2019. The number of DALYs was 1.5 million (95% UI 0.9 to 2.5 million). The percent change in the age-standardized DALY rate decreased from 1990 to 2019 (Tables 1, 2).

In the 5- to 9-year-old group, the trend of the age-standardized DALY rate showed a gradual decline in low-SDI, middle-SDI, high-middle-SDI and low-middle-SDI regions between 1990 and 2019; only high-SDI regions experienced a substantial increase. Nevertheless, the percent change in the age-standardized DALY rate in each SDI region was highest in the high-SDI group and lowest in the low-SDI group (Figure 3B). At the regional level, the age-standardized DALY rate showed intermittent increases and decreases as the SDI increased. The two peaks were at SDIs of 0.55 and 0.65 (Figure 4B). At the national level, age-standardized DALY rates did not change obviously with increasing SDIs, though the age-standardized DALY rate showed a slight decrease from an SDI of 0.55 to 0.65 (Figure 5B).

### The Burden in the 10- to 14-Year-Old Age Group

Nearly 16.4% (2.1 thousand) of children aged 10~14 years old with asthma died due to asthma in 2019. The incidence was ~16.7% (3.7 million), and the percent change in the standardized incidence rate increased by 0.5% (95% UI −1.8 to 4.1%) from 1990 to 2019. The number of DALYs was 1.2 million (95% UI 0.8 to 1.9 million, 24.0%). The percent change in the age-standardized DALY rate decreased from 1990 to 2019 (Tables 1, 2).

In children aged 10~14 years, the change trend of the age-standardized DALY rate with the SDI region was similar to that in those aged 5~9 years, but the age-standardized DALY rate in the low-SDI region was higher than that in the middle-SDI region (Figure 3C). The age-standardized DALY rate at the regional level exhibited an upward trend from an SDI of 0.25 to 0.52 followed by a downward trend from 0.52 to 0.65 (Figure 4C). At the national level, age-standardized DALY rates increased as SDI increased overall, but they decreased gradually from a low SDI to an SDI of 0.55 (Figure 5C).

### The Burden in the 15- to 19-Year-Old Age Group

In 2019, the number of deaths due to asthma among those aged 15 to 19 years was the second largest; the number of incident asthma cases in individuals aged 15 to 19 years was also second largest, at close to 2.4 million. The number of DALYs in those aged 15 to 19 years was smallest (Tables 1, 2). Among those aged 15~19 years, there was no major decreasing trend in the low-SDI region, and the age-standardized DALY rate in the high-SDI region did not change obviously from 1990 to 2019 (Figure 3D). At the regional level, the age-standardized DALY rate decreased sharply between SDIs of 0.25 and 0.62, but it increased rapidly with increasing SDIs above 0.62 (Figure 4D). At the national level, when the SDI was above 0.75, the age-standardized DALY rate increased significantly, but the change was gradual when the SDI was <0.75 (Figure 5D).

### DALYs Attributable to Risk by Age Group and SDI

**Figure 6:**
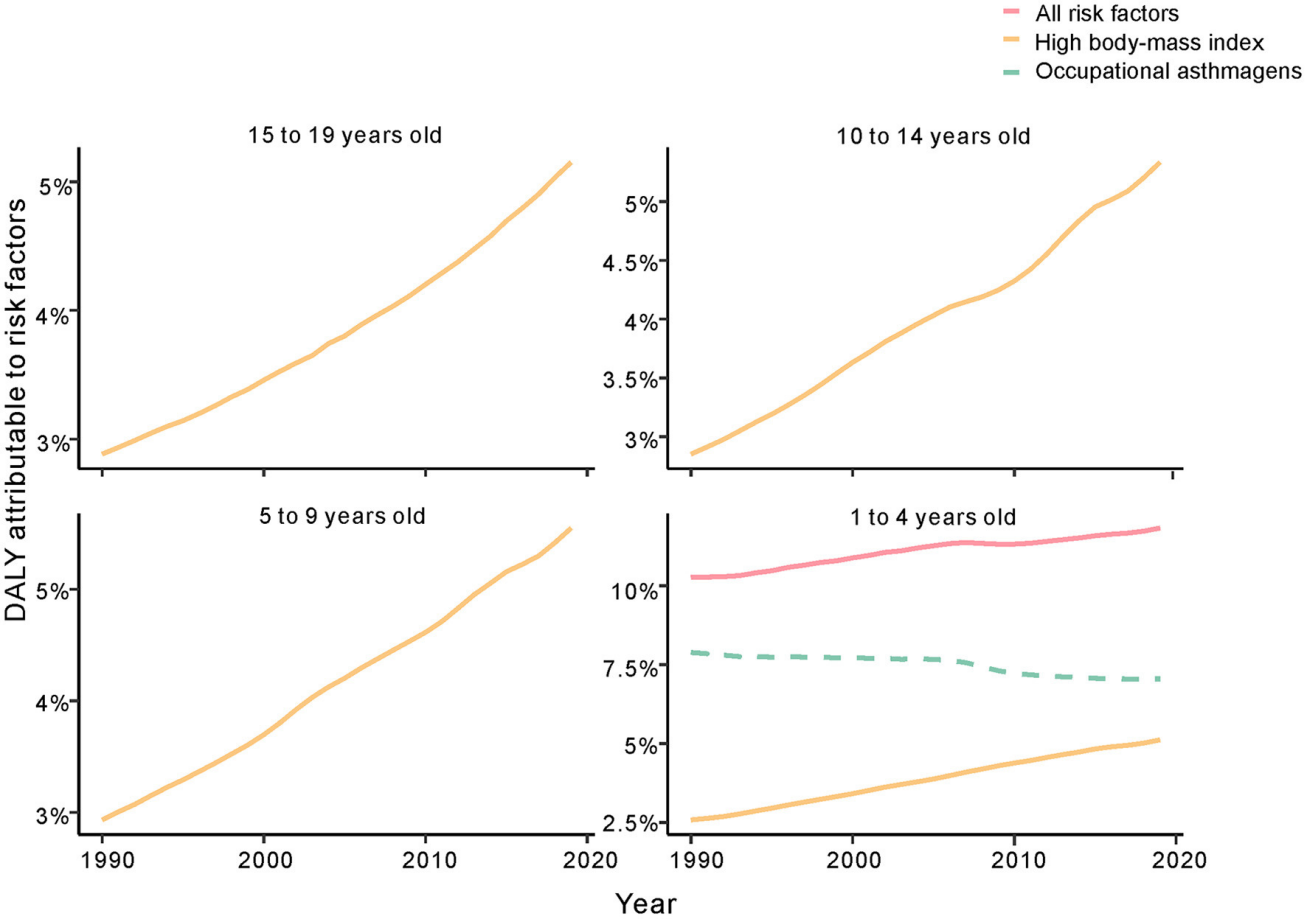
Fraction of age-standardized DALYs attributable to high BMI and occupational asthmagens in childhood asthma by SDI region, 1990–2019. DALYs, disability adjusted life years; SDI, sociodemographic index; BMI, high body mass index.

**Figure 7:**
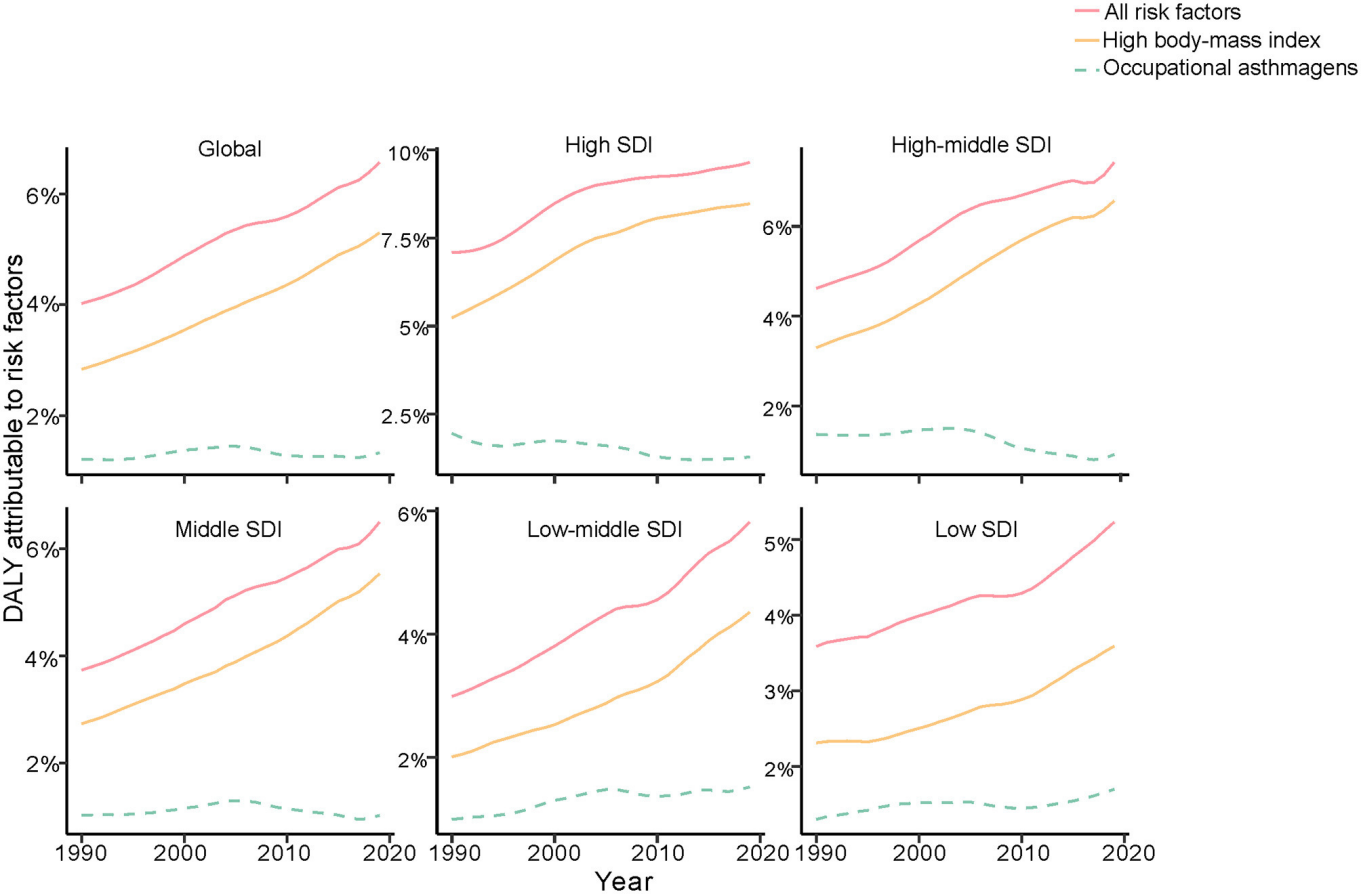
Fraction of age-standardized DALYs attributable to high BMI and occupational asthmagens in childhood asthma by age group, 1990–2019. DALYs, disability adjusted life years; BMI, high body mass index.

According to GBD data, high body mass index (BMI) was an important risk factor for childhood asthma, followed by occupational asthmagens. High BMI was also an independent risk factor for asthma associated with DALYs in the age groups younger than 14 years. The group aged 15 to 19 years had two risk factors, high BMI and occupational asthmagens, with the latter being the most important (Figure 6). In the high-SDI region, the contribution of risk factors to DALYs was largest for high BMI; a similar result was observed in the high-middle-SDI, middle-SDI, low-middle-SDI and low-SDI regions (Figure 7). Additional data can be downloaded from the GBD website.

## Discussion

Asthma is a common respiratory disease in children (19), with an age-standardized incidence rate of 1884.6 per 100 000 in those aged 1 to 4 years in 2019; this was the highest rate among age groups younger than 19 years. The lowest incidence rate was in the 15- to 19-year-old age group, at nearly five times lower than that in the 1- to 4-year-old age group, namely, 387.9 per 100 000 people, in 2019.The mechanism involves exposure to ambient air pollutants, especially in matter of aerodynamic diameters ≤2.5 μm (PM2.5) and ≤10 μm (PM10), SO_2_ and NO_2_ (20, 21). With the reduction in air pollutants, the risk of asthma decreased in those older than 15 years of age. Moreover, the age-standardized mortality rates of childhood asthma decreased sharply, especially in those aged 1 to 4 years. This situation greatly affected the age-standardized DALY rate, with the highest rates in individuals aged 1 to 4 years (264.6 per 100 000) and 5 to 9 years (228.2 per 100 000). Due to the substantial economic and social burdens imposed by pediatric disability along with the significant declines in incidence and mortality rates, our research mainly focused on the age-standardized DALY rate. The general understanding of asthma treatment and management and interpretation of clinical data proves that regular inhaled corticosteroids and other drugs can reduce the incidence and mortality of asthma in children (22).

In addition, age group analysis showed that the age-standardized DALY rate in individuals aged 1 to 4 years decreased in all SDI regions, with the largest decrease in the low-SDI region. However, the age-standardized DALY rate exhibited different trends in other age groups; the age-standardized DALY rate was highest in the high-SDI region and increased from 1990 to 2019 in individuals aged 5 to 9 years, 10 to 14 years and 15 to 19 years. Based on the results, the largest number of DALYs in children with asthma, excluding those aged 1 to 4 years, occurred in the high-SDI region, and the number of DALYs decreased from 1990 to 2019 in the low-SDI region. The level of medical care in the high-SDI region allows for the availability of more health services and for formal drug treatment and prevention recommendations for childhood asthma (23–25). This may explain the decreasing DALY rate in each SDI region and the lowest rate in the high-SDI region in 2019, indicating an inverse relationship between SDI and DALYs. Despite improvements in medical care, the age-standardized DALY rate was still high in the high-SDI region among children older than 5 years. Furthermore, GBD data analysis showed risk factors for asthma to be high BMI and occupational asthmagens, and the most important risk factor was the latter. BMI is calculated by dividing weight (kg) by the square of height (m) (26); it is related to food intake and is an indicator of overweight and obesity. In the high-SDI region, the increase in the age-standardized DALY rate was mainly caused by high BMI in individuals aged over 5 years. Together, children aged over 5 years in the USA spend much time in school away from their parents. Additionally, they encounter new triggers and stressors, such as struggling with asthma in class because of incomplete management. Schools are not tasked with reminding children to take their asthma medication, and self-sufficiency should be emphasized (27). As we age, social influence become less important, and children might forget to take medicine or misunderstand instructions. To avoid embarrassment, children may not take their medication regularly, but severe asthma due to lack of control has a heavy medical burden.

The economic burden of childhood asthma is related to medical care, “medication and loss of productivity among families and societies (28). Furthermore, we should raise awareness about the increasing number of DALYs due to childhood asthma (29), reduce the risk of childhood asthma and adopt formal treatment and management recommendations. In 2019, the number of DALYs due to childhood asthma was highest in the low-SDI region, but the age-standardized DALY rate of childhood asthma was highest in the high-SDI region, with an increase of 3% from 1990 to 2019. This difference stems from slow population growth and the change in the population age structure. Therefore, reducing high BMI is vital in high-SDI regions.

Analyses of the regional associations between SDI and the age-standardized DALY rate indicated that the age-standardized DALY rate in children in high-income North America sharply increased compared with the expected rate from 1990 to 2019. The countries in that region should be given more attention to prevent an increase in DALYs due to childhood asthma. Detailed information for each country revealed that different countries had different age-standardized DALY rates for the target age groups; preventive measures should be taken to reduce the age-standardized DALY rate in specific age groups. For example, Madagascar showed a high age-standardized DALY rate in individuals aged 10 to 19 years; therefore, prevention and treatment recommendations need to be more extensive for individuals in this age group. Cockroaches and their excreta are also related to childhood asthma. Air pollution, socioeconomic status and weather have major impacts on the increasing number of childhood asthma cases (30, 31).

Another risk factor for childhood asthma was occupational asthmagens; regrettably, information about occupational asthmagens was missing for children under 15 years of age. According to the data available, the contribution of occupational asthmagens to the age-standardized DALY rate decreased but remained high; simultaneously, the proportion of children with high BMI increased rapidly from 1990 to 2019, indicating that the effect of high BMI may be greater than that of occupational asthmagens. These results show the importance of high BMI for DALYs among children. The highest age-standardized DALY rate was in the high-SDI region, and we estimated the incidence rates of risk factors for childhood asthma from GBD data. Research indicates that BMI is related to the incidence of childhood asthma via DNA methylation (32). Epidemiological studies (2, 7, 33, 34) have proposed various causes of childhood asthma, including environmental exposures, gene interactions, sensitivity to multiple foods and inhalation of allergens. People with the same ethnic background who live in diverse environments with different environmental conditions have very different incidences. However, whether incidence is related to DALYs has not been reported. Our analysis of childhood asthma data in the GBD database by age group revealed a relationship between DALYs and high BMI. In the high-SDI region, weight control and healthy diets are the main methods to reduce DALYs due to childhood asthma.

The estimates of age-standardized DALY rates in each SDI region in all age groups suggest a slow increase in those aged 1 to 4 years but a sharp increase in those aged 5 to 19 years in the high-SDI region. Although the incidence of high BMI increased sharply in children aged 1 to 4 years from 1990 to 2019, the DALY rate may be influenced by low BMI for the first 4 years of life (32, 35). The age-standardized mortality rate in the low-SDI region declined by 66.4% but was still higher than that in other regions from 1990 to 2019. The age-standardized DALY rate declined significantly in all age groups, and the estimated incidence of risk factors was 18.6 per 100 000 for high BMI and 16.7 per 100 000 for occupational asthmagens. Formal treatment and management, effective drug use and improved clinical care are essential ways to reduce DALYs due to childhood asthma in low-SDI regions (36, 37), but information about risk factors for childhood asthma is limited.

High BMI related to eating habits, lifestyle and food intake can lead to obesity in children (38). Current research shows that children's diets and lifestyle behaviors are associated with high BMI (38–40). In general, childhood obesity is an increasing public health problem worldwide.

## Conclusions

Childhood asthma of the age-standardized DALY rates was increasing in high SDI, especially among those aged 5 to 19 years. However, the change in age-standardized DALY rates gradually decreased in those 1–4 years old. Risk factors included BMI, with a greater risk this risk with a high SDI. Childhood asthma is a widespread chronic disease, and the associated medical and economic burdens remain high. We need to establish recommendations for prevention and treatment, as childhood asthma has received less attention than other chronic diseases, such as childhood leukemia or other childhood cancers and cardiovascular disease. Critical and correct intervention policies require information about childhood asthma cases. Based on the current information, children in high-SDI regions, such as the United States and Canada, should change their diet and lifestyle habits and exercise regularly. Data on additional childhood chronic diseases need to be collected to formulate improved health prevention recommendations, especially regarding risk factors.

## Data Availability Statement

The original contributions presented in the study are included in the article/Supplementary Material, further inquiries can be directed to the corresponding author.

## Author Contributions

DZ wrote the manuscript and analyzed the data. JZ designed the research, downloaded, and explained the generation of related data and graphics. The final manuscript was read and approved by both authors.

## Funding

This research was supported by the Anhui Province Quality Improvement Project.

## Conflict of Interest

The authors declare that the research was conducted in the absence of any commercial or financial relationships that could be construed as a potential conflict of interest.

## Publisher's Note

All claims expressed in this article are solely those of the authors and do not necessarily represent those of their affiliated organizations, or those of the publisher, the editors and the reviewers. Any product that may be evaluated in this article, or claim that may be made by its manufacturer, is not guaranteed or endorsed by the publisher.

## Abbreviations

DALYs: disability-adjusted life years
GBD: Global Burden of Disease
SDI: sociodemographic index
ICD-10: International Classification of Diseases 10th edition
UI: uncertainty interval
CI: confidence interval
BMI: body mass index.
