# Supplementary material for: The Burden of Childhood Asthma by Age Group, 1990–2019: A Systematic Analysis of Global Burden of Disease 2019 Data
Source: Front Pediatr. 2022 Feb 16;10:823399. doi: 10.3389/fped.2022.823399 (PMC8888872; doi:10.3389/fped.2022.823399)
Supplement: Supplementary file 1 [file Table_1.DOCX]

Table s1: Percentage change in age-standardized rates of childhood asthma by region and country, 1990-2019.

|  | **Deaths(95%UI)** | | **Incidence(95%UI)** | | **DALYs(95%UI)** | |
| --- | --- | --- | --- | --- | --- | --- |
|  | **2019 age-standardized rates (per 100,000)** | **Percentage change in age-standardized rates, 1990-2019** | **2019 age-standardized rates (per 100,000)** | **Percentage change in age-standardized rates, 1990-2019** | **2019 age-standardized rates (per 100,000)** | **Percentage change in age-standardized rates, 1990-2019** |
| **Regions** |  |  |  |  |  |  |
| High-income North America | 0.23(0.21 to 0.25) | -30.43 (-36.14 to -23.31) | 2634.6(1901.2 to 3435.32) | 8.03(-5.99 to 23.65) | 510.34(320.81 to 765.9) | 11.42(-3.08 to 29.92) |
| Southern Latin America | 0.07(0.06 to 0.09) | -69.43 (-77.07 to -59.71) | 1235.86(850.44 to 1730.16) | 8.44(-3.99 to 25.18) | 261(155.06 to 425.59) | 8.97(-8 to 30.85) |
| Western Europe | 0.07 (0.06 to 0.08) | -48.3(-55.1 to -37.5) | 909.96(610.75 to 1239.02) | 2.72(-5.48 to 11.11) | 259.67(188.69 to 352.54) | -7.77(-18.07 to 1.39) |
| Australasia | 0.2(0.16 to 0.25) | -68.61 (-74.92 to -59.27) | 1273.16(884.89 to 1725.94) | -18.25(-33.71 to 0.03) | 219.62(132.7 to 358.27) | -27.91(-42.93 to 10.38) |
| High-income Asia Pacific | 0.03(0.02 to 0.04) | -93.92(-95.53 to -90.45) | 984.11(656.63 to 1391.07) | -12.79(-19.04 to 7.09) | 436.86(263.41 to 691.15) | -28.76(-38.01 to 21.02) |
| Eastern Europe | 0.03 (0.03 to 0.04) | -81.86 (-84.7 to -72.81) | 923.3(590.05 to 1330.48) | -6.6(-11.14 to 2.32) | 151.31(85.96 to 254.05) | -13.93(-20.74 to 8.52) |
| East Asia | 0.03 (0.02 to 0.04) | -92.07 (-94.44 to -85.09) | 723.51(463.94 to 1072.88) | 3.31(-0.42 to 7.25) | 121(68.72 to 202.24) | -17.39(-30.72 to 7.95) |
| Central Europe | 0.02(0.02 to 0.03) | -82.25(-85.65 to -77.6) | 993.19(665.66 to 1412.53) | 9.74(-1.9 to 17.48) | 166.67(97.64 to 276.06) | 5.88(-2.24 to 13.32) |
| Central Asia | 0.06 (0.05 to 0.08) | -75.1 (-81.21 to -60.98) | 656.43(417.71 to 946.18) | 5.73(-0.89 to 11.33) | 107.23(62.95 to 176.46) | -5.19(-16.85 to 3.72) |
| Oceania | 2.27 (1.33 to 3.8) | -40.3(-60.89 to -13.4) | 965.56(722.71 to 1258.45) | -10.57(-16.35 to 3.93) | 344.5(238.56 to 479.8) | -30.32(-46.62 to 12.14) |
| Western Sub-Saharan Africa | 0.8 (0.56 to 1.13) | -57.0 (-68.35 to -39.73) | 912.1(618.57 to 1285.67) | -8.37(-12.16 to 5.13) | 208.96(143.86 to 300.18) | -31.64(-42.59 to 19.52) |
| Southern Sub-Saharan Africa | 0.54 (0.42 to 0.68) | -67.43 (-75.74 to -54.63) | 882.16(592.51 to 1258.18) | -1.03(-6.3 to 5.67) | 216.12(137.93 to 334.35) | -28.02(-42.2 to 14.18) |
| Eastern Sub-Saharan Africa | 1.28 (0.91 to 1.91) | -69.78 (-80.21 to -38.21) | 1234.39(854.71 to 1686.06) | -13.24(-19.74 to 8.26) | 317.51(222.44 to 445.82) | -47.22(-59.97 to 25.22) |
| Central Sub-Saharan Africa | 1.13 (0.74 to 1.67) | -73.26 (-83.61 to -42.42) | 936.98(659.93 to 1276.99) | -14.43(-22.6 to 6.47) | 246.32(168.47 to 341.55) | -54.06(-69.25 to 26.12) |
| Southeast Asia | 1.2(0.94 to 1.41) | -67.58(-75.5 to -47.22) | 297.32(201.26 to 428.14) | -0.87(-5.89 to 3.69) | 82.1(55.43 to 120.72) | -44.53(-56.24 to 25.81) |
| North Africa and Middle East | 0.43(0.33 to 0.58) | -77.16(-83.91 to -62.55) | 964.98(659.64 to 1337.18) | -2.04(-9.56 to 4.15) | 200.61(129.49 to 306.69) | -37.03(-50.91 to 20.87) |
| Tropical Latin America | 0.21(0.17 to 0.28) | -80.98(-86.35 to -70.26) | 2052.03(1344.34 to 2825.94) | -10.98(-18.38 to 2.56) | 376.77(229.54 to 614.34) | -28(-39.13 to 19.43) |
| Central Latin America | 0.22(0.17 to 0.31) | -86.38(-90.24 to -76.68) | 1246.27(837.27 to 1735.97) | -15.21(-23.55 to 9.05) | 237.11(146.58 to 377.44) | -41.62(-53.27 to 30.47) |
| Andean Latin America | 0.24(0.16 to 0.38) | -93.87(-96.55 to -85.74) | 1814.37(1277.02 to 2482.05) | -22.28(-35.16 to 12.39) | 371.56(224.21 to 610.67) | -54.97(-67.87 to 40.3) |
| Caribbean | 2.42(1.17 to 4.03) | -50.99(-68.48 to -24.89) | 1962.17(1440.37 to 2594.76) | -6.75(-11.84 to 2.44) | 584.24(387.67 to 824.88) | -29.28(-43.73 to 13.87) |
| South Asia | 0.29(0.24 to 0.35) | -73.05(-79.46 to -58.78) | 2634.6(1901.2 to 3435.32) | -23.19(-29.55 to 17.02) | 510.34(320.81 to 765.9) | -47.18(-59.25 to 31.22) |
| **Countries** |  |  |  |  |  |  |
| Uruguay | 0.18(0.13 to 0.24) | -53.08(-70.34 to -32.34) | 1189.92(832.35 to 1625.12) | -3.11(-14.08 to 8.63) | 256.23(156.04 to 412.92) | -9.66(-23.28 to 3.94) |
| Chile | 0.04(0.03 to 0.05) | -59.36(-71.81 to -41.82) | 1032.25(698.81 to 1450.19) | -0.95(-14.47 to 15.97) | 204.55(256.55 to 342.14) | 1.46(-15.33 to 22.23) |
| Argentina | 0.07(0.05 to 0.1) | -72.82(-81.13 to -61.21) | 1308.93(903.43 to 1830.5) | 11.69(-2.2 to 31.13) | 280.73(356.63 to 459.32) | 11.74(-6.86 to 36.07) |
| United Kingdom | 0.19(0.16 to 0.21) | -62.49(-66.99 to -57.83) | 1385.53(931.77 to 1931.11) | -18.2(-23.36 to 13.47) | 335.09(456.81 to 525.54) | -27.01(-32.95 to 21.55) |
| Switzerland | 0.04(0.03 to 0.05) | -78.42(-85.09 to -65.57) | 942.08(639.01 to 1297.07) | 1.39(-9.14 to 12.74) | 242.76(556.36 to 400.67) | -5.11(-16.61 to 6.9) |
| Sweden | 0.05(0.04 to 0.06) | -67.05(-75.54 to -56.41) | 1081.92(710.73 to 1545.28) | 5.82(-3.18 to 14.97) | 267.78(656.72 to 442.86) | -0.04(-10.23 to 10.98) |
| Spain | 0.04(0.03 to 0.06) | -78.08(-85.13 to -67.4) | 795.65(530.48 to 1096.69) | 21.75(-3.91 to 48.34) | 170.93(756.12 to 277.48) | 4.77(-14.72 to 29.08) |
| Portugal | 0.02(0.01 to 0.03) | -92.67(-95.44 to -87.78) | 1058.14(710.52 to 1464.85) | 6.19(-10.69 to 34.37) | 305.65(856.94 to 497.6) | 6.04(-14.79 to 44.07) |
| Norway | 0.04(0.03 to 0.04) | -76.22(-80.86 to -70.86) | 1180.87(786.73 to 1658.07) | 6.79(-1.62 to 11.76) | 268.06(956.35 to 431.37) | 1.83(-4.69 to 7.5) |
| Netherlands | 0.04(0.03 to 0.05) | -75.94(-83.47 to -65.71) | 521.66(380.48 to 709.05) | -0.56(-13.13 to 24.55) | 207.41(1056.29 to 330.13) | -8.08(-19.4 to 11.99) |
| Malta | 0.05(0.03 to 0.07) | -67.22(-77.71 to -53.03) | 920.78(635.84 to 1266.63) | 7.53(-6.05 to 26.28) | 232.07(1156.49 to 380.79) | 6.61(-11.15 to 27.34) |
| Luxembourg | 0.07(0.05 to 0.1) | -78.79(-85.62 to -68.1) | 916.19(617.13 to 1254.05) | -9.1(-17.88 to 0.87) | 243.85(1256.9 to 396.06) | -16.67(-25.8 to 8.59) |
| Italy | 0.02(0.02 to 0.03) | -87.79(-90.25 to -81.74) | 814.39(517.6 to 1143.42) | 48.88(-31.75 to 70.7) | 155.42(1356.34 to 251.56) | 13.94(-1.69 to 28.19) |
| Israel | 0.06(0.04 to 0.08) | -82.82(-88.85 to -72.32) | 791.42(532.51 to 1076.88) | -1.37(-9.12 to 7.46) | 185.18(1456.87 to 299.94) | -15.35(-25.98 to 5.92) |
| Ireland | 0.08(0.06 to 0.11) | -77.58(-84.51 to -66.89) | 980.71(673.02 to 1326.55) | -12.18(-23.43 to 0.83) | 264.76(1556.33 to 421.85) | -22.58(-34.68 to 11.69) |
| Iceland | 0.05(0.03 to 0.06) | -74.36(-83.39 to -61.61) | 1249.43(840.48 to 1741.59) | -11.51(-29.37 to 3.34) | 270.74(1656.28 to 445.4) | -21.54(-41.63 to 5.38) |
| Greece | 0.02(0.02 to 0.03) | -67.53(-76.84 to -54.86) | 771.42(517.98 to 1045.34) | 21.02(-1.64 to 60.26) | 188.79(1756.94 to 315.24) | 24.26(-0.6 to 82.9) |
| Germany | 0.04(0.03 to 0.06) | -87.47(-91.4 to -78.89) | 696.19(465.7 to 948.39) | -4.6(-17.92 to 7.9) | 162.07(1856.96 to 262.45) | -16(-30.92 to 0.97) |
| France | 0.05(0.03 to 0.06) | -81.22(-86.38 to -72.18) | 901.14(613.94 to 1240.64) | 3.49(-11.24 to 22.25) | 233.84(1956.22 to 379.19) | 0.17(-18.75 to 23) |
| Taiwan (Province of China) | 0.03(0.02 to 0.04) | -85.31(-90.54 to -77.47) | 976.17(644.82 to 1420.34) | 24.29(-5.54 to 52.77) | 185.14(2056.63 to 302.83) | 15.95(-7.14 to 51.23) |
| Finland | 0.03(0.02 to 0.04) | -71.93(-80.34 to -60.21) | 762.05(516.8 to 1039) | -2.9(-13.04 to 9.02) | 204.24(2156.46 to 336.09) | -2.97(-15.61 to 9.53) |
| Denmark | 0.04(0.03 to 0.06) | -79.83(-86.83 to -69.36) | 840.54(567.72 to 1153.29) | 2.06(-6.29 to 13.28) | 189.77(2256.27 to 312.27) | -7.17(-19.02 to 3.49) |
| Cyprus | 0.02(0.01 to 0.03) | -73.41(-83.82 to -54.71) | 927.7(629.64 to 1272.02) | 1.92(-6.39 to 9.68) | 228.3(2356.94 to 373.68) | 0.06(-8.86 to 10.09) |
| Belgium | 0.05(0.04 to 0.07) | -81.3(-86.88 to -74.21) | 736.75(496.07 to 1006.82) | 3.19(-12.5 to 20.77) | 175.96(2456.34 to 281.68) | -6.73(-23.36 to 13.53) |
| Austria | 0.03(0.02 to 0.04) | -80.87(-86.75 to -73.04) | 788.68(540.09 to 1071.63) | 7.93(-7.95 to 27.01) | 192.18(2556.43 to 311.1) | 3.15(-13.76 to 24.72) |
| Andorra | 0.07(0.05 to 0.1) | -73.98(-84.79 to -54.83) | 845.05(563.43 to 1177) | -4.16(-12.2 to 3.04) | 230.76(2656.36 to 373.86) | -10.92(-20.86 to 2.65) |
| New Zealand | 0.27(0.22 to 0.33) | -66.53(-72.91 to -57.6) | 1455.12(979.85 to 2042.28) | -17.18(-26.23 to 8.25) | 365.79(2756.73 to 580.17) | -34.3(-41.73 to 26.39) |
| Australia | 0.19(0.15 to 0.24) | -69.01(-76.51 to -57.69) | 1237.37(863.11 to 1670.04) | -18.32(-36.72 to 4.06) | 450.84(2856.39 to 715.65) | -26.87(-44.17 to 4.83) |
| Democratic People's Republic of Korea | 0.13(0.09 to 0.19) | -75.72(-85.93 to -52.95) | 798.51(536.46 to 1142.98) | -10.36(-17.56 to 2.51) | 149.3(2956.12 to 244.3) | -19.68(-34.07 to 5.79) |
| Singapore | 0.04(0.02 to 0.08) | -93.58(-96.11 to -86.2) | 937.8(636.93 to 1317.61) | 4.48(-11.6 to 25.71) | 175.04(3056.63 to 285.75) | -25.31(-41.86 to 7.06) |
| Republic of Korea | 0.02(0.01 to 0.03) | -96.48(-98.07 to -93.57) | 855.08(582.15 to 1197.68) | 11.26(-1.29 to 26.46) | 165.39(3156.42 to 277.38) | -11.01(-30.03 to 7.36) |
| Japan | 0.03(0.02 to 0.04) | -92.88(-94.65 to -88.97) | 1043.87(684.56 to 1492.74) | -20.2(-25.58 to 15.25) | 193.04(3256.16 to 315.97) | -34.42(-41.73 to 28.31) |
| Brunei Darussalam | 0.1(0.07 to 0.15) | -80.56(-87.59 to -67.65) | 887.2(605.78 to 1242.17) | -5.54(-12.41 to 2.59) | 176.96(3356.98 to 286.76) | -15.13(-26.36 to 4.76) |
| Ukraine | 0.04(0.02 to 0.06) | -60.56(-77.28 to -18.49) | 1118.69(701.91 to 1623.84) | -5.5(-12.57 to 3.13) | 189.74(3456.45 to 316.46) | -7.21(-16.43 to 2.09) |
| Russian Federation | 0.04(0.03 to 0.04) | -84.28(-86.55 to -77.53) | 874.06(559.43 to 1266.32) | -6.97(-10.86 to 3.5) | 141.31(3556.88 to 234.21) | -16.59(-23.8 to 11.01) |
| Republic of Moldova | 0.01(0.01 to 0.02) | -85.61(-90.64 to -72.76) | 823.21(527.34 to 1189.04) | -3.28(-10.34 to 5.67) | 140.3(3656.39 to 232.53) | -1.93(-11.23 to 8.02) |
| Lithuania | 0.02(0.01 to 0.03) | -85.58(-90.73 to -72.94) | 829.64(535.84 to 1175.16) | 18.61(-1.26 to 50.42) | 136.75(3756.66 to 224.5) | 14.14(-6.57 to 44.69) |
| China | 0.03(0.02 to 0.04) | -92.71(-94.97 to -85.78) | 718.23(459.47 to 1066.17) | 3.28(-0.66 to 7.27) | 119.46(3856.55 to 199.89) | -17.91(-31.21 to 8.49) |
| Latvia | 0.02(0.01 to 0.03) | -90.36(-94.03 to -82.91) | 942.81(611.91 to 1343.1) | 9.28(-7.8 to 35.52) | 157.71(3956.04 to 259.79) | 2.71(-16.93 to 28.55) |
| Estonia | 0.02(0.01 to 0.03) | -84.98(-90.89 to -77) | 690.92(445.24 to 977.16) | -4.65(-19.97 to 13.01) | 110.94(4056.28 to 183.94) | -9.89(-28.17 to 8.25) |
| Belarus | 0.02(0.01 to 0.03) | -89.44(-93.91 to -75.13) | 984.78(622.52 to 1421.76) | -1.54(-8.95 to 6.03) | 163.12(4156.69 to 271.98) | -9.14(-19.6 to -0.06) |
| Slovenia | 0.01(0.01 to 0.02) | -74.08(-82.96 to -58.61) | 1029.99(700.54 to 1457.62) | 4.17(-2.99 to 12.27) | 175.42(4256.09 to 290.22) | 0.97(-8.66 to 11.26) |
| Slovakia | 0.03(0.02 to 0.04) | -72.11(-81.98 to -58.71) | 760.96(515.78 to 1075.53) | 6.34(-0.05 to 13.36) | 123.44(4356.04 to 205.86) | 0.18(-9.57 to 10.1) |
| Serbia | 0.04(0.03 to 0.06) | -87.33(-91.36 to -81.02) | 720.71(488.35 to 995.67) | 0.36(-6.27 to 6.89) | 120.63(4456.09 to 200.12) | -15.64(-27.14 to 5) |
| Sudan | 0.8(0.5 to 1.22) | -82.1(-90.45 to -46.54) | 1023.59(695.82 to 1437.64) | -4.46(-18.37 to 6.39) | 241.16(4556.65 to 359.6) | -56.64(-73.67 to 21.69) |
| Romania | 0.01(0.01 to 0.02) | -87.84(-92.89 to -77.27) | 963.89(646.64 to 1350.69) | 20.67(-2.86 to 57.86) | 164.14(4656.22 to 269.69) | 23.27(-1.59 to 70.86) |
| Poland | 0.02(0.02 to 0.02) | -86.57(-89.09 to -81.71) | 1269.07(841.36 to 1822.82) | 11.04(-0.92 to 19.92) | 211.97(4756.57 to 354.92) | 9.22(-0.58 to 16.3) |
| Montenegro | 0.01(0.01 to 0.01) | -59.13(-72.17 to -43.08) | 865.7(591.65 to 1214.97) | 2.38(-3.67 to 9.66) | 145.81(4856.74 to 241.12) | 3.53(-6.07 to 13.78) |
| North Macedonia | 0.02(0.01 to 0.03) | -78.54(-86.8 to -66.14) | 971.87(668.81 to 1369.97) | -0.9(-6.93 to 5.59) | 164.4(4956.06 to 271.73) | -4.35(-13.42 to 4.86) |
| Hungary | 0.03(0.02 to 0.05) | -75.36(-83.51 to -62.59) | 830.07(563.82 to 1169.02) | 2.53(-4.65 to 9.65) | 139.73(5056.06 to 231.76) | -4.27(-14.17 to 5.73) |
| Czechia | 0.04(0.03 to 0.05) | -74.62(-82.65 to -63.28) | 797.12(539.09 to 1123.64) | 7.01(-0.64 to 14.84) | 130.44(5156.39 to 213.69) | -3.43(-13.99 to 6.57) |
| Croatia | 0.01(0.01 to 0.02) | -80.27(-86.44 to -71.6) | 894.78(605.53 to 1250.57) | 0.75(-6.48 to 6.49) | 153.31(5256.47 to 255.06) | -1.34(-10.88 to 7.83) |
| Bulgaria | 0.02(0.01 to 0.03) | -80.21(-87.23 to -70.53) | 867.33(581.78 to 1204.89) | 4.73(-1.81 to 11.96) | 145.38(5356.44 to 235.79) | 1.02(-9.12 to 10.66) |
| Bosnia and Herzegovina | 0.02(0.01 to 0.02) | -61.29(-74.91 to -39.67) | 955.46(651.75 to 1351.75) | 3.08(-1.81 to 9.07) | 167.24(5456.13 to 276.25) | 3.96(-4.73 to 13.81) |
| South Sudan | 1.77(1.09 to 2.78) | -67.5(-82.8 to -32.65) | 1200.36(835.96 to 1621.14) | -14.51(-21.94 to 7.06) | 354.9(5556.61 to 496.47) | -50.01(-67.84 to 22.98) |
| Albania | 0.09(0.04 to 0.15) | -72.21(-83.5 to -55.21) | 708.1(482.26 to 988.18) | 19.2(-3.92 to 46.44) | 120.5(5656.32 to 195.21) | 2.6(-20.24 to 32.93) |
| United States Virgin Islands | 0.16(0.11 to 0.23) | -65.29(-77.94 to -46.52) | 1842.45(1298.98 to 2488.65) | 7.57(-0.19 to 15.71) | 370.35(5756.05 to 614.78) | 6.12(-3.79 to 15.86) |
| Tuvalu | 0.55(0.37 to 0.83) | -87.07(-93.08 to -70.45) | 927.7(622.58 to 1302.94) | 7.3(-6.74 to 22.42) | 207.79(5856.73 to 316.93) | -57.09(-74.13 to 29.72) |
| Tokelau | 0.3(0.19 to 0.46) | -81.78(-89.78 to -67.48) | 1047.37(701.61 to 1481.65) | 3.93(-6.75 to 18.14) | 200.18(5956.31 to 316.06) | -31.83(-52.24 to 10.34) |
| Uzbekistan | 0.05(0.03 to 0.09) | -68.42(-80.04 to -49.85) | 723.99(464.68 to 1049.62) | -0.42(-12.2 to 9.49) | 119.56(6056.98 to 197.09) | -3.89(-17.55 to 8.73) |
| Turkmenistan | 0.08(0.06 to 0.11) | -91.28(-94.3 to -84.37) | 644.61(408.31 to 940.6) | -5.3(-13.85 to 4.08) | 106.26(6156.07 to 172.54) | -41.91(-58.5 to 25.51) |
| San Marino | 0.03(0.02 to 0.04) | -49.14(-70.71 to -12.7) | 840.8(563.32 to 1165.24) | 1.35(-5.72 to 9) | 219.26(6256.02 to 360.26) | -0.28(-8.68 to 9.79) |
| Saint Kitts and Nevis | 0.23(0.15 to 0.32) | -56.16(-74.64 to -31.56) | 1908.76(1361.78 to 2573.96) | 2.91(-3.18 to 12.61) | 403.08(6356.02 to 645.6) | 2.74(-7.13 to 12.52) |
| Tajikistan | 0.01(0.01 to 0.02) | -22.66(-54.6 to 28.9) | 634.82(400.24 to 919.09) | 4.66(-1.96 to 12.39) | 98.85(6456.55 to 161.73) | 8.41(-2 to 20.36) |
| Puerto Rico | 0.18(0.12 to 0.26) | -68.65(-79.6 to -52.47) | 2205.13(1581.47 to 2949.99) | -10.07(-16.83 to 2.09) | 525.38(6556.27 to 831.25) | -7.81(-17.72 to 2.61) |
| Palau | 0.23(0.14 to 0.34) | -68.73(-84.33 to -34.05) | 912.92(620.05 to 1273.01) | -7.37(-13.63 to 0.35) | 183.42(6656.07 to 284.47) | -20.5(-36.82 to 5.72) |
| Mongolia | 0.06(0.03 to 0.1) | -88.08(-93.99 to -66.09) | 647.43(414.67 to 943.04) | 11.77(-4.27 to 20.1) | 101.02(6756.02 to 164.64) | -21.75(-50.45 to 1.22) |
| Northern Mariana Islands | 0.13(0.09 to 0.19) | -53.1(-69.06 to -28.68) | 916.84(630.77 to 1278.55) | -15.15(-22.93 to 5.43) | 186.68(6856.12 to 308.9) | -9.74(-19.56 to 2.86) |
| Niue | 0.48(0.27 to 0.78) | -52.01(-71.89 to -22.54) | 966.42(655.48 to 1313.7) | -3.27(-10.35 to 4.62) | 213.26(6956.91 to 328.15) | -15.67(-30.36 to 1.28) |
| Kyrgyzstan | 0.02(0.02 to 0.06) | -77.5(-86.83 to -47.75) | 716.71(460.19 to 1039.95) | 4.11(-2.24 to 11.44) | 114.13(7056.69 to 190.02) | -0.51(-11.43 to 9.21) |
| Nauru | 0.98(0.56 to 1.47) | -59.09(-73.51 to -34.94) | 925.96(626.17 to 1305.08) | -1.8(-11.84 to 8.47) | 234.13(7156.01 to 337.51) | -32.15(-49.24 to 12.74) |
| Monaco | 0.04(0.03 to 0.06) | -54.41(-70.26 to -29.32) | 853.91(571.38 to 1175.28) | 1.07(-6.29 to 8.47) | 220.68(7256.53 to 362.82) | -0.61(-8.69 to 8.15) |
| Kazakhstan | 0.07(0.05 to 0.1) | -67.61(-78.79 to -50.87) | 528.41(335.44 to 749.69) | 15.66(-8.61 to 23.41) | 84.54(7356.25 to 140.46) | -2.24(-16.61 to 10.96) |
| Guam | 0.1(0.07 to 0.14) | -47.15(-65.83 to -21.29) | 1065.65(719.5 to 1517.23) | -5.76(-11.23 to 1.02) | 194.77(7456.77 to 323.11) | -7.74(-16.13 to 0.83) |
| Georgia | 0.34(0.22 to 0.47) | -39.02(-57.68 to -11.79) | 659.67(424.28 to 957.76) | 14.93(-1.22 to 38.36) | 132.85(7556.5 to 200.68) | -1.71(-20.49 to 21.16) |
| Greenland | 0.16(0.1 to 0.28) | -74.28(-84.29 to -56.5) | 1750.24(1240.11 to 2432.74) | -24.19(-31.8 to 15.97) | 320.83(7656.23 to 517.41) | -29.23(-38.51 to 20.01) |
| Azerbaijan | 0.04(0.03 to 0.06) | -55.74(-73.3 to -27.5) | 617.52(394.73 to 886.64) | 5.18(-2.28 to 13.54) | 102.36(7756.69 to 168.2) | 5.11(-6.14 to 16.67) |
| Armenia | 0.01(0.01 to 0.01) | -52.7(-71.61 to -22.09) | 646.35(415.76 to 947.55) | 7.61(-1.78 to 14.74) | 103.4(7856.21 to 171.89) | 10(-0.81 to 22.32) |
| Cook Islands | 0.06(0.03 to 0.11) | -87.64(-94.24 to -74.75) | 1030.21(696 to 1434.31) | -3.98(-11.05 to 3.18) | 190.72(7956.42 to 308.3) | -16.42(-28.8 to 6.51) |
| Bermuda | 0.12(0.08 to 0.16) | -64.08(-75.8 to -46.39) | 1946.92(1354.96 to 2617.26) | 2.82(-3.41 to 10.12) | 406.31(8056.89 to 655.65) | 6.72(-3.43 to 16.15) |
| Vanuatu | 1.13(0.77 to 1.73) | -37.56(-58.75 to -7.49) | 857.14(561.68 to 1197.91) | 2.03(-14.93 to 20.41) | 231.13(8156.45 to 340.38) | -16.24(-35.53 to 5.47) |
| American Samoa | 0.29(0.2 to 0.41) | -60.14(-75.22 to -37.59) | 938.71(640.58 to 1302.98) | -6.94(-14.75 to 3) | 192.67(8256.5 to 301.66) | -13.88(-27.9 to 0.11) |
| Tonga | 0.28(0.18 to 0.42) | -52.59(-72.05 to -25.05) | 1016.22(682.66 to 1413.04) | -3.91(-9.35 to 3.99) | 199.21(8356.74 to 313.25) | -14.84(-26.15 to 4.39) |
| Solomon Islands | 0.79(0.56 to 1.09) | -46.44(-64.68 to -18.96) | 885.65(611.16 to 1260.48) | 5.78(-4.72 to 16.96) | 207.4(8456.13 to 297.92) | -17.19(-34.29 to 0.82) |
| Samoa | 0.41(0.26 to 0.61) | -70.77(-82.91 to -53.07) | 787.96(533.55 to 1104.36) | -2.81(-11.23 to 11.01) | 169.37(8556.22 to 262.26) | -31.88(-48.17 to 15.5) |
| Papua New Guinea | 2.59(1.4 to 4.46) | -44.38(-65.5 to -11.89) | 995.01(753.63 to 1296.83) | -14.58(-21.59 to 6.45) | 375.58(8656.44 to 538.39) | -35.63(-52.93 to 14.31) |
| Micronesia (Federated States of) | 0.72(0.39 to 1.03) | -67.95(-82.61 to -51.79) | 816.44(562.12 to 1115.9) | -2.97(-15.6 to 11.38) | 197.52(8756.1 to 286.89) | -37.04(-53.9 to 18.93) |
| Marshall Islands | 0.9(0.6 to 1.31) | -41.05(-59.94 to -12.22) | 805.24(557.41 to 1082.48) | -7.99(-19.51 to 3.14) | 204.18(8856.5 to 285.7) | -21.52(-37.15 to 5.68) |
| Kiribati | 2.45(1.66 to 3.73) | -57.96(-72.62 to -31.25) | 869.76(647.91 to 1133.76) | -25.13(-32.22 to 17.4) | 331.55(8956.03 to 444.84) | -50.06(-63.23 to 29.21) |
| Fiji | 1.6(1.15 to 2.16) | -44.34(-64.6 to -17.15) | 761.82(536.19 to 1053.48) | -13.87(-22.67 to 3.68) | 249.39(9056.79 to 329.69) | -32.48(-49.58 to 14.61) |
| Togo | 0.62(0.36 to 0.99) | -63.5(-79.08 to -38.62) | 963.86(637.41 to 1364.5) | -5.25(-16.59 to 5.36) | 204.41(9156.19 to 304.78) | -30.87(-47.19 to 14) |
| Sierra Leone | 1.33(0.71 to 2.27) | -57.68(-78.4 to -18.68) | 840.44(567.9 to 1177.17) | -13.26(-22.46 to 3.3) | 240.71(9256.14 to 345.43) | -41.3(-60.61 to 14.01) |
| Senegal | 0.55(0.33 to 0.85) | -75.25(-86.45 to -51.79) | 698.18(461.63 to 986.46) | -1.87(-12.7 to 8.35) | 150.99(9356.49 to 224.68) | -47.23(-64.73 to 25.41) |
| Sao Tome and Principe | 0.6(0.39 to 0.89) | -78.62(-86.95 to -64.35) | 924.32(627.01 to 1267.43) | -24.96(-36.41 to 13.24) | 201.38(9456.68 to 295.76) | -53.91(-65.43 to 40.01) |
| Nigeria | 0.6(0.38 to 0.87) | -58.56(-71.7 to -33.28) | 1037.27(707.13 to 1461.14) | -14.05(-17.98 to 10.44) | 215.14(9556.69 to 321.85) | -29.7(-41.68 to 16.14) |
| Niger | 1.7(0.88 to 2.99) | -62.82(-80.29 to -25.38) | 859.7(583.35 to 1212.34) | -14.51(-23.52 to 2.75) | 270.75(9656.63 to 400.62) | -49.61(-67.25 to 20.73) |
| Mauritania | 0.25(0.14 to 0.4) | -75.67(-87.07 to -53.23) | 1113.8(743.59 to 1570.01) | -5.86(-15.9 to 3.15) | 211.39(9756.48 to 331.51) | -23.26(-38.99 to 7.99) |
| Mali | 0.53(0.29 to 1.31) | -45.32(-67.04 to -5.88) | 673.89(453.39 to 951.33) | 9.81(-0.72 to 18.58) | 139.76(9856.17 to 218.19) | -13.14(-37.89 to 7.68) |
| Liberia | 0.57(0.28 to 1.21) | -85.15(-93.46 to -62.64) | 785.83(524.46 to 1108.46) | -16.85(-29.32 to 5.95) | 171.14(9956.85 to 263.57) | -63.95(-78.05 to 40.42) |
| Guinea-Bissau | 0.95(0.56 to 1.47) | -71.44(-84.98 to -46.23) | 806.97(541.81 to 1106.97) | -8.98(-19.37 to 1.05) | 200.77(10056.54 to 287.17) | -50.77(-67.04 to 27.95) |
| Guinea | 1.55(0.91 to 2.47) | -56.59(-75.47 to -21.6) | 951.41(662.1 to 1369.25) | -6.84(-16.61 to 3.5) | 275.01(10156.4 to 393.7) | -39.14(-58.9 to 13.09) |
| Ghana | 0.64(0.33 to 0.99) | -60.96(-76.79 to -34.81) | 685.75(458.85 to 960.55) | 2.42(-4.97 to 11.1) | 158.15(10256.46 to 228.03) | -32.02(-50.9 to 8.74) |
| Gambia | 0.55(0.32 to 0.92) | -62.89(-80.72 to -24.5) | 801.09(529.53 to 1105.54) | -4.81(-13.23 to 6.23) | 169.49(10356.37 to 255.55) | -31.43(-52.12 to 9.13) |
| Côte d'Ivoire | 0.75(0.44 to 1.17) | -51.98(-72.55 to -17.7) | 909.88(612.56 to 1288.35) | 1.96(-7.98 to 10.91) | 202.66(10456.18 to 298.45) | -22.31(-41.16 to 2.54) |
| Chad | 1.46(0.85 to 2.33) | -44.24(-68.35 to -1.64) | 724.41(496.25 to 1026.25) | 4.61(-6.03 to 16.19) | 226.4(10556.34 to 320.87) | -28.64(-52.21 to 1.94) |
| Cabo Verde | 0.11(0.07 to 0.18) | -82.99(-91.15 to -59.9) | 817.98(547.46 to 1149.46) | -1.54(-10.39 to 7.77) | 142.54(10656.11 to 228.65) | -19.9(-37.7 to 4.45) |
| Cameroon | 0.66(0.37 to 1.05) | -53.33(-74.57 to -20.1) | 731.45(485.27 to 1035.48) | 2.77(-8.64 to 14.59) | 167.44(10756.29 to 245.37) | -23.57(-43.16 to 2.43) |
| Burkina Faso | 1.43(0.76 to 2.57) | -39.29(-69.18 to 23.29) | 856.27(584.31 to 1212.79) | 0.65(-10.14 to 9.46) | 250.3(10856.67 to 369.15) | -23.56(-47.72 to 11.95) |
| Benin | 1.07(0.59 to 1.76) | -56.54(-76.06 to -25.33) | 825.74(557.84 to 1156.05) | -7.02(-17.19 to 2.55) | 213.72(10956.96 to 307.98) | -36.17(-55.12 to 14.75) |
| Viet Nam | 0.31(0.19 to 0.44) | -69.91(-81.52 to -45.42) | 762.15(518.81 to 1055.83) | 10(-1.89 to 18.62) | 157.77(11056.15.15) | -20.48(-40.85 to 0.78) |
| Zimbabwe | 0.6(0.37 to 0.86) | 5.64(-39.24 to 82.17) | 461.25(309.06 to 646.16) | 1.41(-4.34 to 9.89) | 117.03(11156.16 to 163.7) | 3.72(-18.96 to 30.29) |
| Eswatini | 1.04(0.69 to 1.42) | -54.16(-71.88 to -25.15) | 834.08(557.62 to 1148.47) | -13.08(-22.68 to 2.73) | 224.2(11256.4 to 309.62) | -34.71(-50.91 to 17.26) |
| South Africa | 0.43(0.33 to 0.55) | -78.43(-85.05 to -66.35) | 1096.32(706.63 to 1597.94) | -0.4(-6.61 to 7.46) | 261.58(11356.88 to 419.04) | -30.45(-47.24 to 13.71) |
| Namibia | 0.65(0.36 to 0.94) | -62.44(-80.3 to -20.66) | 552.61(367.73 to 765.87) | -0.05(-10.07 to 9.34) | 136.96(11456.74 to 196.11) | -38.91(-57.87 to 8.58) |
| Lesotho | 1.5(0.92 to 2.26) | -30.19(-55.65 to 9.14) | 348.12(241.64 to 464.77) | -2.17(-9.47 to 6.82) | 170.14(11556.11 to 237.78) | -23.24(-45.42 to 4.83) |
| Botswana | 1(0.39 to 1.65) | -34.33(-73.32 to 12.32) | 734.66(466.86 to 1084.69) | 34.51(-11.75 to 76.47) | 201.36(11656.43 to 293.6) | -1.65(-35.71 to 34.86) |
| Zambia | 0.97(0.62 to 1.62) | -76.92(-88.62 to -35.85) | 853.03(582.4 to 1168.41) | 8.16(-3.87 to 21.71) | 213.81(11756.28 to 310.43) | -54.96(-73.35 to 15.08) |
| Uganda | 1.29(0.77 to 2.17) | -68.7(-83.33 to -37.99) | 1419.04(968.91 to 1969.45) | -13.97(-25.74 to 2.51) | 357.62(11856.67 to 512.38) | -43.58(-60.68 to 21.47) |
| Timor-Leste | 1.88(0.71 to 2.87) | -80.98(-93.77 to -33.73) | 842.28(614.65 to 1131.24) | -21.79(-32.09 to 10.91) | 300.18(11956.61 to 413.47) | -70.54(-84.49 to 29.09) |
| United Republic of Tanzania | 1.35(0.79 to 2.38) | -47.67(-72.71 to 23.17) | 1648.19(1142.76 to 2240.37) | -0.65(-8.26 to 7.85) | 415.62(12056.42 to 604.3) | -18.98(-40.2 to 8.86) |
| Somalia | 2.87(1.7 to 5.04) | -55.05(-75.63 to -9.61) | 1249.11(915.18 to 1700.52) | -17.54(-25.86 to 9.32) | 443.31(12156.9 to 641.05) | -44.98(-63.45 to 15.51) |
| Seychelles | 0.26(0.19 to 0.35) | -44.64(-60.09 to -23.09) | 773.22(525.35 to 1105.25) | 12.96(-5.65 to 20.5) | 153.52(12256.89 to 240.39) | 0.6(-12.73 to 12.22) |
| Rwanda | 1.47(0.83 to 2.69) | -78.73(-89.7 to -43.95) | 1834.42(1282.62 to 2472.98) | -25.59(-34.39 to 16.15) | 492.61(12356.34 to 726.12) | -54.59(-69.93 to 34.22) |
| Mozambique | 1.19(0.7 to 2.01) | -73.64(-87.91 to -13.45) | 1257.83(880.93 to 1710.35) | -9.59(-19.02 to 1.27) | 308.22(12456.41 to 447.54) | -50.27(-68.96 to 11.03) |
| Mauritius | 0.74(0.55 to 1.02) | -67.41(-77.32 to -51.83) | 762.54(530.77 to 1036.4) | -14.76(-23.75 to 5.59) | 199.71(12556.95 to 288.66) | -42.68(-54.15 to 29.97) |
| Malawi | 1.06(0.67 to 1.62) | -74.81(-86.62 to -28) | 1115.86(761.86 to 1502.81) | -10.88(-21.91 to 1.4) | 280.54(12656.31 to 397.77) | -50.47(-68.55 to 17.46) |
| Madagascar | 2.64(1.54 to 4.99) | -69.45(-82.71 to -36.7) | 1572.85(1151.35 to 2099.34) | -37.99(-45.26 to 30.52) | 501.84(12756.37 to 729.79) | -59.28(-69.63 to 39.42) |
| Kenya | 0.68(0.45 to 1.25) | -49.69(-66.78 to -5.53) | 909.38(603.88 to 1268.96) | -12.03(-15.16 to 8.84) | 202.57(12856.85 to 302.32) | -25.01(-38.29 to 6.46) |
| Thailand | 0.33(0.24 to 0.44) | -67.54(-77.31 to -52.2) | 876.88(594.79 to 1228.77) | 7.52(-6.15 to 24.47) | 191.44(12956.51 to 299.81) | -15.71(-33.68 to 2.41) |
| Ethiopia | 0.81(0.56 to 1.31) | -80.45(-88.74 to -49.5) | 1010.79(668.91 to 1416.48) | -9.95(-17.49 to 2.64) | 226.54(13056.09 to 324.61) | -56.53(-70.87 to 28.64) |
| Eritrea | 1.47(0.91 to 2.27) | -74.28(-87.4 to -46.49) | 1209.74(849.39 to 1632.33) | -12.6(-21.52 to 3.43) | 330.46(13156.81 to 464.49) | -53.73(-70.95 to 28.89) |
| Djibouti | 1(0.53 to 1.69) | -62.96(-79.92 to -33.51) | 1255.31(872.5 to 1725.01) | -18.17(-27.59 to 9.45) | 296.27(13256.86 to 426.97) | -39.97(-55.13 to 23.24) |
| Comoros | 0.91(0.48 to 1.49) | -69.33(-84.79 to 17.14) | 1209.02(832.64 to 1634.04) | -11.24(-22 to -0.04) | 286.97(13356.67 to 414.18) | -39.72(-58.95 to 1.79) |
| Burundi | 2.06(1.19 to 3.73) | -73.41(-87.77 to -34.69) | 1279.92(894.01 to 1720.1) | -19.49(-27.71 to 10.72) | 382.05(13456.23 to 566.13) | -58.59(-74.92 to 28.42) |
| Gabon | 0.36(0.21 to 0.54) | -75.8(-85.84 to -54.87) | 887.05(603.58 to 1212.3) | -7.01(-15.39 to 0.88) | 177.81(13556.56 to 274.84) | -35.64(-53.73 to 15.1) |
| Equatorial Guinea | 0.34(0.17 to 0.58) | -91.4(-96.08 to -80.03) | 926.86(636.87 to 1272.64) | -17.91(-30.38 to 6.75) | 188.25(13656.73 to 292.1) | -63.31(-78.39 to 40.61) |
| Democratic Republic of the Congo | 1.14(0.7 to 1.75) | -72.3(-84.91 to -31.21) | 911.29(643.73 to 1230.4) | -12.88(-21.7 to 4.45) | 241.55(13756.14 to 341.57) | -53.06(-71.17 to 20.14) |
| Congo | 0.57(0.34 to 0.9) | -74.74(-85.81 to -54.58) | 1048.86(707.18 to 1445.4) | -6.54(-16.73 to 2.75) | 224.86(13856.86 to 337.89) | -40.19(-58.14 to 21.49) |
| Sri Lanka | 0.34(0.24 to 0.51) | -82.1(-88.76 to -72.06) | 719.99(495.65 to 999.6) | 10.47(-0.94 to 23.3) | 154.45(13956.34 to 238.97) | -41.08(-57.51 to 23.4) |
| Central African Republic | 2.88(1.49 to 5.02) | -35.84(-63.3 to 9.61) | 967.48(706.51 to 1284.95) | -14.42(-21.08 to 6.89) | 397.57(14056.53 to 594.05) | -29.18(-51.3 to 0.13) |
| Angola | 0.98(0.52 to 1.52) | -81.78(-90.93 to -58.03) | 989.45(688.34 to 1334.57) | -22.53(-33.04 to 12.49) | 243.27(14156.74 to 346.81) | -63.91(-79.2 to 37.96) |
| Pakistan | 0.44(0.32 to 0.59) | -50.26(-68.91 to -16.82) | 278.6(188.71 to 400.64) | -20.29(-26.81 to 14.37) | 80.41(14256.15 to 112.4) | -37.83(-54.23 to 17.76) |
| Nepal | 0.16(0.1 to 0.36) | -82.7(-92.62 to -39.63) | 154.76(106.77 to 216.2) | -14.86(-28.54 to 2.56) | 37.23(14356.37 to 56.07) | -64.57(-80.89 to 23.05) |
| India | 0.27(0.21 to 0.33) | -74.28(-81.24 to -56.3) | 316.74(210.82 to 458.31) | -24.14(-30.67 to 17.36) | 86.94(14456.54 to 130.14) | -44.95(-58.89 to 27.37) |
| Bhutan | 0.28(0.16 to 0.46) | -78.03(-89.83 to -17.13) | 207.4(147.98 to 289.58) | -31.8(-44.1 to 19.83) | 56.49(14556.87 to 83.05) | -64.25(-79.52 to 27.4) |
| Bangladesh | 0.26(0.18 to 0.44) | -83.98(-89.99 to -69.1) | 196.09(133.83 to 271.75) | -22.12(-34.11 to 10.65) | 53.25(14656.9 to 76.7) | -69.42(-79.19 to 54.02) |
| Afghanistan | 0.94(0.61 to 1.41) | -70.59(-82.66 to -43.24) | 989.09(699.8 to 1344.39) | 4.86(-3.59 to 14.02) | 235.89(14756.3 to 339.83) | -42.14(-59.84 to 16.69) |
| Philippines | 2.27(1.69 to 2.73) | -59(-69.48 to -34.64) | 1415.59(1013.91 to 1945.53) | -20.87(-24.36 to 16.93) | 437.85(14856.87 to 585.48) | -44.65(-54.27 to 28.72) |
| Yemen | 0.65(0.42 to 0.99) | -72.25(-83.94 to -41.03) | 980.34(661.94 to 1359.12) | -8.8(-17.52 to 0.04) | 218.46(14956.96 to 324.46) | -40.76(-57.34 to 17.92) |
| United Arab Emirates | 0.24(0.15 to 0.41) | -76.02(-85.6 to -61.05) | 1323.01(901.19 to 1832.12) | -6.84(-14.76 to 3.05) | 277.15(15056.27 to 437.95) | -15.92(-30.26 to 3.69) |
| Turkey | 0.12(0.08 to 0.25) | -90.09(-94.58 to -80.5) | 1009.87(713.53 to 1369.64) | -8.68(-17.76 to 1.95) | 201.34(15156.99 to 316.16) | -33.99(-49.48 to 19.19) |
| Tunisia | 0.16(0.1 to 0.24) | -82.38(-89.2 to -70.29) | 962.02(649 to 1348.11) | 6.78(-3.54 to 20.14) | 181.33(15256.5 to 290.91) | -19.35(-36.89 to 2.49) |
| Syrian Arab Republic | 1.36(0.81 to 1.79) | -38.82(-59.98 to -5.53) | 871.51(614.36 to 1159.46) | -5.34(-16.17 to 6.89) | 266.01(15356.23.23) | -18.87(-36.35 to 0.31) |
| Saudi Arabia | 0.15(0.11 to 0.21) | -80.61(-87.48 to -68.85) | 846.36(573.99 to 1143.07) | 23.05(-10.61 to 43.39) | 159.95(15456.64 to 254.71) | -6.48(-31.6 to 20) |
| Qatar | 0.12(0.08 to 0.17) | -81.85(-88.49 to -70.62) | 1027.95(693.33 to 1458.64) | -5.75(-14.22 to 4.12) | 187.71(15556.43 to 303.35) | -19.86(-33.46 to 6.7) |
| Oman | 0.03(0.02 to 0.04) | -72.19(-82.07 to -55.21) | 1141.77(762.77 to 1604.5) | 29.6(-12.27 to 55.75) | 197.13(15656.28 to 328.02) | 32.26(-8.14 to 61.5) |
| Myanmar | 2.2(1.39 to 3.36) | -73.64(-86.72 to -43.81) | 526.01(383.18 to 705.95) | -3.44(-12.33 to 6.8) | 264.52(15756.32 to 370.67) | -65.84(-80.21 to 35.72) |
| Palestine | 0.1(0.07 to 0.18) | -82.54(-89.81 to -68.58) | 986.65(673.5 to 1376.38) | 5.44(-3.58 to 14.57) | 179.06(15856.25 to 290.14) | -7.58(-24.44 to 8.34) |
| Morocco | 0.3(0.18 to 0.47) | -72.88(-83.75 to -49.98) | 826.69(564.12 to 1129.23) | 8.99(-3.15 to 22.02) | 164.23(15956.42 to 257.08) | -22.19(-42.3 to 0.5) |
| Libya | 0.22(0.14 to 0.33) | -68.16(-81.13 to -48.03) | 937.41(636.68 to 1282.03) | -4.13(-11.55 to 4.84) | 189.95(16056.41 to 302.54) | -13.23(-27.56 to 0.32) |
| Lebanon | 0.11(0.07 to 0.17) | -71.83(-83.21 to -54.33) | 1164.57(781.35 to 1643.36) | 7(-4.23 to 17.35) | 211.98(16156.61 to 344.61 to 344) | -0.24(-16.97 to 14.05) |
| Kuwait | 0.13(0.09 to 0.18) | -78.51(-85.94 to -66.13) | 1179.53(779.53 to 1671.03) | -1.15(-12.1 to 10.36) | 222.55(16256.53 to 361.66) | -12.77(-27.89 to 0.91) |
| Jordan | 0.14(0.1 to 0.2) | -70.06(-79.94 to -56.61) | 1113.78(749.93 to 1560.26) | 5.56(-2.52 to 15.24) | 215.74(16356.39 to 353.27) | -0.32(-13.08 to 11.87) |
| Iraq | 0.19(0.13 to 0.26) | -82.93(-89.34 to -71.84) | 989.7(674.66 to 1389.94) | -10.7(-19.57 to 0.19) | 188.96(16456.86 to 302.56) | -30.93(-46.3 to 16.1) |
| Iran (Islamic Republic of) | 0.17(0.13 to 0.25) | -81.24(-87.45 to -69.31) | 977.08(647.45 to 1372.47) | -4.6(-10.08 to 0.32) | 172.48(16556.33 to 275.3) | -27.78(-43.04 to 15.53) |
| Egypt | 0.49(0.3 to 0.9) | -87.57(-93.34 to -73.05) | 901.4(609.43 to 1235.38) | -9.97(-21.18 to 1.06) | 192.61(16656.62 to 292.44) | -61.03(-74.24 to 42.22) |
| Bahrain | 0.27(0.21 to 0.35) | -71.31(-79.44 to -59.1) | 1008.11(693.49 to 1380.58) | -7.27(-15.46 to 1.57) | 206.63(16756.94 to 324.19) | -18.99(-32.23 to 6.86) |
| Maldives | 0.3(0.2 to 0.45) | -92.54(-96.15 to -84.04) | 853.31(587.59 to 1219.81) | -29.76(-43.08 to 17.38) | 168.5(16856.64 to 260.04) | -69.46(-80.98 to 50.89) |
| Algeria | 0.24(0.17 to 0.37) | -78.26(-85.65 to -66.02) | 982.79(660.12 to 1381.82) | 20.39(-7.67 to 35.62) | 184.63(16956.6 to 295.11) | -17.04(-36.65 to 3) |
| Paraguay | 0.1(0.07 to 0.14) | -82.29(-89.44 to -69.86) | 1979.92(1345.13 to 2743.03) | 4.8(-3.98 to 15.81) | 383.04(17056.2 to 624.12) | 2.7(-12.77 to 16.77) |
| Brazil | 0.22(0.17 to 0.29) | -80.86(-86.3 to -70.12) | 2054.97(1344.59 to 2832.2) | -11.34(-18.8 to 2.64) | 376.52(17156.37 to 615.15) | -28.68(-39.85 to 19.97) |
| Venezuela (Bolivarian Republic of) | 0.28(0.19 to 0.41) | -78.53(-86.47 to -62.14) | 1412.61(967.84 to 1944.46) | -17.16(-28.33 to 8.27) | 279.24(17256.12 to 442.25) | -35.42(-47.72 to 23.49) |
| Panama | 0.45(0.31 to 0.63) | -73.3(-83.32 to -53.26) | 1637.83(1115.36 to 2236.84) | -12.79(-25.91 to 0.44) | 340.5(17356.82 to 523.09) | -33.52(-46.92 to 19.07) |
| Nicaragua | 0.37(0.19 to 0.64) | -86.71(-93.49 to -73.23) | 1369.52(938.09 to 1906.36) | -35.34(-46.67 to 24.74) | 278.25(17456.61 to 439.23) | -57.78(-68.15 to 45.9) |
| Mexico | 0.15(0.12 to 0.22) | -82.92(-88.02 to -72.72) | 1140.22(744.84 to 1605.31) | -3(-10.6 to 3.43) | 206.85(17556.43 to 335.89) | -24.21(-37.98 to 12.89) |
| Malaysia | 0.18(0.12 to 0.26) | -79.76(-87.35 to -67.37) | 777.6(531.78 to 1106.39) | 9.51(-4.57 to 30.71) | 150.67(17656.96 to 238.06) | -18.31(-38.03 to 1.84) |
| Honduras | 0.79(0.42 to 1.29) | -88.5(-94.36 to -76.5) | 1425.6(987.98 to 1930.95) | -33.15(-44.54 to 23.73) | 321.5(17756.83 to 488.16) | -67.72(-77.52 to 54.82) |
| Guatemala | 0.44(0.3 to 0.61) | -94.51(-96.53 to -89.49) | 1135.53(784.65 to 1575.87) | -45.24(-56.98 to 35.34) | 229.74(17856.28 to 356.89) | -78.46(-85.31 to 68.3) |
| El Salvador | 0.13(0.07 to 0.21) | -96.78(-98.47 to -90.68) | 1665.04(1143.16 to 2292.41) | -35.06(-47.17 to 22.96) | 325.81(17956.58 to 512.76) | -64.78(-74.93 to 51.79) |
| Costa Rica | 0.09(0.06 to 0.15) | -67.08(-79 to -48.73) | 1709.69(1164.65 to 2345.99) | -14.36(-26.93 to 3.38) | 337.27(18056.01 to 542.07) | -14.87(-29.43 to 1.36) |
| Colombia | 0.11(0.06 to 0.21) | -85.91(-92.29 to -74.06) | 1285.18(880.13 to 1782.94) | -14.62(-22.23 to 7.71) | 236.91(18156.91 to 378.23) | -29.24(-40.05 to 20.4) |
| Peru | 0.12(0.07 to 0.21) | -95.51(-98.04 to -89.91) | 1820.43(1264.63 to 2516.56) | -23.61(-39 to 11.04) | 367.54(18256.9 to 622.71) | -49.41(-64.85 to 33.73) |
| Ecuador | 0.16(0.1 to 0.3) | -96.12(-97.8 to -82.75) | 1708.02(1200.02 to 2359.57) | -22(-34.2 to 10.47) | 345.22(18356.43 to 564.9) | -57.55(-69.68 to 38.87) |
| Bolivia (Plurinational State of) | 0.65(0.33 to 1.01) | -91.71(-96.25 to -80.4) | 1942.21(1388.77 to 2654.09) | -18.85(-29.92 to 8.38) | 416.49(18456.99 to 632.03) | -63.96(-78.01 to 44.63) |
| Lao People's Democratic Republic | 1.92(1.23 to 2.84) | -83.76(-91.16 to -57.11) | 600.93(436.19 to 816.38) | -18.61(-27.07 to 9.45) | 251.93(18556.38 to 348.61) | -77.27(-86.45 to 50.51) |
| Trinidad and Tobago | 0.67(0.46 to 0.93) | -56.77(-72.42 to -34.64) | 1709.56(1202.29 to 2322.12) | 10.49(-2.81 to 23.76) | 375.99(18656.11 to 577.09) | -4.66(-20.88 to 12.29) |
| Suriname | 0.46(0.32 to 0.63) | -50.4(-67.58 to -11.03) | 1892.78(1358.86 to 2561.58) | 2.96(-4.93 to 11.83) | 404.35(18756.57 to 630.87) | -2.91(-15.48 to 10.13) |
| Saint Vincent and the Grenadines | 0.35(0.25 to 0.47) | -48.06(-66.21 to -22.77) | 1882.93(1326.4 to 2526.71) | 2.57(-4.38 to 9.95) | 398.01(18856.88 to 641.49) | -1.04(-11.32 to 9.24) |
| Saint Lucia | 0.63(0.45 to 0.87) | -49.99(-68.21 to -22.59) | 1920.49(1379.23 to 2548.25) | -5.49(-13.61 to 3.32) | 445.1(18956.51 to 682.16) | -9.53(-20.96 to 1.02) |
| Jamaica | 0.46(0.33 to 0.62) | -69.19(-80.02 to -51.15) | 1874.35(1333.23 to 2508.4) | -15.45(-25.24 to 6.89) | 416.8(19056.23 to 665.17) | -26.71(-39.62 to 14.84) |
| Haiti | 5.53(2.11 to 9.81) | -62.38(-78.62 to -29.48) | 2246.27(1694.39 to 2913.52) | -16.21(-23.89 to 8.46) | 898.83(19156.21 to 1298.48) | -49.35(-65 to 23.73) |
| Guyana | 0.7(0.5 to 0.96) | -40.34(-61.13 to -8.84) | 1947.3(1427.52 to 2578.38) | 1.49(-6.8 to 9.08) | 427.03(19256.82 to 652.28) | -4.18(-15.79 to 7.78) |
| Grenada | 0.26(0.18 to 0.35) | -56.74(-71.91 to -32.61) | 2093.14(1488.76 to 2844.21) | -1.2(-8.75 to 7.04) | 450.54(19356.14 to 724.1) | -1.21(-11.89 to 9.1) |
| Dominican Republic | 1.05(0.67 to 1.55) | -78.69(-87.8 to -65.34) | 1479.68(1058.24 to 2024.4) | -6.62(-18.6 to 6.12) | 342.7(19456.97 to 503.16) | -50.75(-65.08 to 33.72) |
| Dominica | 0.78(0.53 to 1.1) | 12.68(-32.58 to 74.47) | 1983.06(1409.76 to 2613.38) | 4.57(-4.48 to 13.88) | 472.69(19556.76 to 698.69) | 13.81(-3.49 to 26.62) |
| Indonesia | 1.04(0.79 to 1.26) | -73.34(-80.75 to -55.5) | 893.02(622.57 to 1214.32) | -1.87(-10.33 to 6.02) | 236.46(19656.05 to 329.67) | -50.48(-62.79 to 30.34) |
| Cuba | 0.18(0.13 to 0.26) | -57.2(-70.05 to -36.53) | 2129.27(1494.3 to 2857.16) | 3.02(-5.36 to 11.26) | 454.52(19756.57 to 738.37) | 2.76(-8.1 to 12.73) |
| Belize | 0.51(0.38 to 0.67) | -65.36(-76.26 to -48.82) | 1860.89(1304.16 to 2468.91) | -11.57(-21.85 to 2.15) | 408.77(19856.03 to 643.68) | -21.85(-34.09 to 8.8) |
| Barbados | 0.27(0.19 to 0.37) | -38.35(-58.05 to -7.21) | 1949.2(1382.91 to 2630.06) | 10.06(-3.36 to 27.4) | 420.52(19956.16 to 675.98) | 12.91(-4.22 to 34.38) |
| Bahamas | 0.24(0.16 to 0.33) | -39.48(-61.91 to -5.56) | 1835.4(1281.51 to 2464.76) | 3.45(-4.42 to 11.81) | 395.65(20056.18 to 633.46) | 6.91(-3.39 to 16.72) |
| Antigua and Barbuda | 0.18(0.13 to 0.26) | -11.44(-43.46 to 37.65) | 1847.88(1308.13 to 2491.94) | -1.15(-8.16 to 6.45) | 385.21(20156.59 to 615.08) | 6.13(-3.04 to 15.66) |
| United States of America | 0.25(0.22 to 0.27) | -27.6(-33.65 to -20.38) | 2753.44(1986.34 to 3593.95) | 8.56(-6.75 to 24.95) | 533.79(20256.02 to 800.2) | 12.23(-3.19 to 32.22) |
| Canada | 0.08(0.06 to 0.1) | -69.6(-77.79 to -58.02) | 1440.78(982 to 1957.53) | -4.01(-23.12 to 5.54) | 274.82(20356.95 to 456.93) | -4.85(-19.94 to 5.44) |
| Cambodia | 0.68(0.49 to 0.98) | -78.42(-87.25 to -44.03) | 731.14(505.7 to 1032.6) | 8.09(-2.4 to 22.49) | 176.81(20456.76 to 256.07) | -51.84(-67.91 to 16.09) |
